# Healthcare access inequalities in Quilombola rural and riverside communities in the Brazilian Amazon: Associations with social, organizational, and territorial factors

**DOI:** 10.1371/journal.pone.0355489

**Published:** 2026-08-10

**Authors:** Leanna Silva Aquino, Ellen Mara Fernandes da Silva, Julianne Figueiredo Costa Sousa, Ednaldo Pereira Maranhão, Victoria Valentim Aguiar, Cesar Ferreira Fernandes Filho, Daliane Ferreira Marinho, Sheyla Mara Silva de Oliveira, Tatiane Costa Quaresma, Valney Mara Gomes Conde, Marcos Manoel Honorato, Veridiana Barreto do Nascimento, Guilherme Augusto Barros Conde, Franciane de Paula Fernandes, Lívia de Aguiar Valentim

**Affiliations:** 1 Academic of the State University of Pará – UEPA, Santarém, Pará, Brasil; 2 Academic of the Metropolitan College of Pará – FAMETRO, Santarém, Pará, Brasil; 3 Academic of the University of Amazonia – UNAMA, Santarém, Pará, Brasil; 4 Assistant professor State University of Pará – UEPA, Santarém, Pará, Brasil; 5 Assistant professor Federal University of West Para- UFOPA, Santarém, Pará, Brasil; 6 PhD in Sciences at the University of São Paulo (USP), Assistant Professor of the Nursing Course at the University of the State of Pará, Santarém, Pará, Brasil; Universidade Federal do Espirito Santo, BRAZIL

## Abstract

**Background:**

Equitable access to healthcare services is a core principle of Brazil’s Unified Health System and remains a persistent challenge in territories marked by social and geographic inequalities, such as the Brazilian Amazon. In this region, vast distances, logistical barriers, and socioeconomic disparities converge to limit the effectiveness of public health policies.

**Objective:**

The present study aimed to examine individual, organizational, and territorial factors associated with self-reported healthcare access among quilombola rural and riverside communities in Santarém, Brazilian Amazon, using conventional statistical and machine-learning approaches.

**Methods:**

This cross-sectional, community-based cross-sectional survey included 512 adults from nine Amazonian communities. Data were collected through structured interviews and analyzed using Python-based computational workflows. Supervised and unsupervised algorithms, multilevel logistic modeling, and principal component analysis (PCA) were employed. Five predictive models (Decision Tree, Random Forest, Gradient Boosting, Logistic Ridge, and Logistic Lasso) were compared using AUC, accuracy, sensitivity, and specificity, with performance reported for cross-validation in the training set and the held-out test set. A Social Vulnerability Index (SVI) was derived from PCA.

**Results:**

Access and problem-resolution rates varied among communities, ranging from 42.9% to 100% and from 54.5% to 100%, respectively. Predictive discrimination was limited in the held-out test set (AUC 0.482–0.586), indicating poor generalizability for individual-level prediction. At the community level (n ≥ 10), the SVI was negatively correlated with access (r ≈ −0.53).

**Conclusions:**

Healthcare access in the Brazilian Amazon reflects intertwined territorial and social constraints. Computational approaches can complement conventional analyses by integrating multi-level information to support equity-oriented planning in remote settings.

## Introduction

Access to healthcare services is a core pillar for consolidating Brazil’s Unified Health System (SUS) and for ensuring the constitutional right to health [1,2]. Despite major institutional and regulatory advances, persistent regional and social inequalities still constrain the effective realization of this right, particularly in peripheral and hard-to-reach territories [3]. In the Brazilian Amazon, long distances, population dispersion, seasonal mobility constraints, and uneven health infrastructure produce a distinctive set of challenges, in which territorial vulnerability converges with enduring socioeconomic disparities [4,5].

Evidence accumulated over recent decades indicates that healthcare access in the Amazon is shaped not only by the nominal presence of services and professionals, but also by a network of interrelated determinants, including income, educational attainment, mobility conditions, occupational profile, and community belonging [6]. These factors influence patterns of service use and, consequently, the timeliness and continuity of care [7,8]. In rural and riverside settings, geographic distance and transportation costs are frequently compounded by infrastructural deficiencies and the precarious condition of transit routes, resulting in access that is both unequal and intermittent [9].

Beyond geography and material conditions, access in the Amazon is also marked by historical and social processes that structure opportunities and constraints in everyday life. Quilombola communities, for instance, have faced long-standing patterns of territorial exclusion and limited public investment, which may translate into barriers to timely care, reduced service availability, and greater dependence on irregular transportation routes [10,11]. In this context, “community belonging” is not merely a demographic attribute; it often reflects lived experiences of discrimination, institutional invisibility, and unequal distribution of resources that can shape care-seeking trajectories and interactions with health services [11,12].

At the same time, access is mediated by how services are organized and delivered in the territory. In remote areas, primary healthcare is frequently the main entry point to SUS, but its reach and continuity may be compromised by workforce instability, logistical constraints, and variability in service provision across communities [11]. In the Amazon, where rivers and seasonal cycles can define mobility, the organization of care, including outreach activities, the presence of community health workers, and the availability of transport support, becomes a decisive component of access, operating alongside individual socioeconomic conditions and territorial factors [13].

Clarifying these multiple determinants is essential for informing policies that are more equitable and responsive to regional specificities and local health dynamics [14]. However, conventional statistical approaches may face constraints in heterogeneous contexts such as the Amazon, where exposures and barriers are unevenly distributed and may interact across levels [15]. In these settings, relationships among variables can be non-linear, conditional, and mutually reinforcing, which can hinder the identification of complex risk profiles and combined effects using strictly parametric frameworks.

In this scenario, machine learning methods have gained visibility in public health research as complementary tools for exploring multifactorial phenomena. By leveraging algorithms capable of handling high-dimensional data and flexible functional forms, these approaches can support the identification of patterns and interactions associated with healthcare access and utilization [16]. The use of supervised models may help estimate the probability of an individual obtaining care, while unsupervised techniques can assist in characterizing population subgroups according to shared vulnerability profiles and barriers related to service use [17,18]. Importantly, when applied to health access, these strategies should be interpreted as analytical instruments that support the identification of risk profiles and the exploration of interactions, rather than as substitutes for conceptual frameworks grounded in social epidemiology and health equity [11,13].

In the Amazon, computational approaches are particularly relevant because they facilitate the integration of individual, community, and territorial attributes within a single analytical framework. This is critical in regions where territory is not merely a geographic backdrop, but an active determinant of living conditions, mobility, and the organization of health services [19]. By combining social, organizational, and contextual information, these techniques may offer additional perspectives on access dynamics in historically marginalized populations, helping to reveal inequalities that can remain underexplored in more aggregated analyses [20]. Nevertheless, there remains a need for analyses grounded in primary data and focused on specific communities, capable of articulating social determinants with territorial and organizational dimensions in a coherent model of access.

Given this context, the present study aimed to examine individual, organizational, and territorial factors associated with self-reported healthcare access among quilombola rural and riverside communities in Santarém, Brazilian Amazon, using conventional statistical and machine-learning approaches.

## Methodology

A community-based cross-sectional survey with a quantitative approach was conducted in quilombola rural and riverside communities in the municipality of Santarém, western Pará, within the Brazilian Amazon. This study was reported according to the Strengthening the Reporting of Observational Studies in Epidemiology (STROBE) guidelines. The target population comprised residents aged 18 years or older, of both sexes, who voluntarily agreed to participate. A total of 512 individuals were interviewed.

The municipality includes 13 officially recognized quilombola communities. Of these, only one community has a local primary healthcare unit providing low-complexity services. The remaining communities must travel to the urban area to access healthcare, covering distances ranging from approximately 10–45 kilometers, by fluvial and/or road transportation, depending on seasonal and geographic conditions. Even in the community with a local primary healthcare unit, individuals requiring specialized or higher-complexity care must travel approximately 45 kilometers to the urban referral center. Due to logistical constraints related to geographic dispersion, river navigation conditions, and accessibility, nine communities were included in the present study.

The sample size was determined using a finite population formula based on the total quilombola population of the municipality (N = 4,363), of whom 2,133 reside in officially recognized territories. Assuming a 95% confidence level and a 5% margin of error, the calculation (𝑛 = 𝑁 / (1 + 𝑁·𝐸²)) indicated a minimum required sample of approximately 326 participants. The calculated sample size was used to define the minimum number of participants required for the overall survey. However, recruitment was not based on probability sampling proportional to the population of each community. Therefore, the sample-size calculation should not be interpreted as guaranteeing statistical representativeness of all quilombola residents or individual communities.

Participant recruitment was community-based and non-probabilistic, including residents who were present and available during fieldwork. This strategy may have introduced selection bias because availability and willingness to participate may be associated with employment, mobility, health status, engagement with community organizations, and previous experiences with healthcare services. Therefore, the observed access estimates may differ from those of residents who were absent, less accessible, or unwilling to participate.

Data were collected through structured, face-to-face interviews conducted by trained researchers and undergraduate students. A standardized questionnaire was applied to obtain information on sociodemographic characteristics (age, sex, education, household income, occupation, and housing conditions), territorial characteristics (community of residence), and variables related to healthcare needs and use. The instrument used in this study was the same validated questionnaire applied in Valentim’s doctoral thesis [11]. Interviews were conducted individually within each community, in a private and confidential setting, to ensure participants’ comfort and reduce social desirability bias.

The primary outcome was healthcare access operationalized as self-reported realized access, defined as having received healthcare when the participant perceived that care was needed during the previous 12 months. This measure captures one specific dimension of access, the successful receipt of care following a perceived need and should not be interpreted as a comprehensive assessment of healthcare accessibility. It does not distinguish all stages between health need, perceived need, care-seeking, service availability, utilization, and adequacy of care, nor does it capture unmet needs that were not recognized or translated into demand. Accordingly, the outcome is referred to throughout the manuscript as “self-reported healthcare access” or “self-reported realized access.”

In the study setting, realized access may be influenced by territorial mobility, transport availability, organization of primary care, referral pathways, and the capacity of the healthcare network to respond to perceived needs. These dimensions were examined as contextual and organizational characteristics but were not assumed to be fully represented by the primary outcome measure. A secondary outcome was problem resolution, defined as the participant’s report that the health problem was resolved after receiving care.

Because resolution occurs logically after access, this variable was not treated as a predictor of access in predictive models, in order to avoid reverse causality and potential data leakage. Instead, resolution was analyzed separately among the subgroup of participants who reported having accessed care, as an outcome reflecting the perceived effectiveness of the care received.

All questionnaires were entered into a structured database and screened for completeness and internal consistency. The database contained 517 records; five observations with missing information for the primary outcome were excluded. Thus, 512 participants with valid information for healthcare access were included in the analytical sample. Missing values in covariates were handled conservatively: categories such as “does not know” were retained as explicit levels when applicable (e.g., household income), and records with missing values in variables required for a given model were excluded only for that specific analysis. The final sample size used in each model is reported in the corresponding tables and figures.

Data were analyzed using Python 3.11, with pandas for data handling, scikit-learn for machine learning workflows, and statsmodels for regression-based inference. No custom software was developed. All analyses were conducted using standard Python package. Descriptive analyses included absolute and relative frequencies for categorical variables and means with standard deviations for continuous variables, to characterize the sample and assess data consistency.

For explanatory variables, complete-case analysis was adopted, and the effective sample size varied slightly across models depending on covariate availability. Categorical variables were encoded using appropriate dummy (one-hot) encoding procedures, and no imputation was performed for missing covariates to avoid introducing model-dependent assumptions. Continuous variables included in the PCA were standardized prior to extraction to ensure comparability of scales. Data preprocessing steps were applied exclusively to the training set during predictive modeling to prevent data leakage, with transformations subsequently applied to the test set.

Machine learning methods were applied as an analytical strategy to explore patterns and determinants of healthcare access. Predictors included individual sociodemographic variables, territorial context (community of residence), and service-related characteristics collected prior to, or contemporaneously with, the access outcome. Variables that are conceptually downstream of access, particularly problem resolution and satisfaction with care, were excluded from access prediction models to prevent leakage and to preserve temporal coherence.

For supervised learning, the following algorithms were implemented: Decision Tree (CART), penalized logistic regression (Ridge and Lasso), Random Forest, and Gradient Boosting. Categorical variables were encoded using one-hot encoding, and continuous variables were standardized when required by the algorithm. The dataset was partitioned into training (80%) and test (20%) sets using a stratified split to preserve the outcome distribution. Model development and hyperparameter tuning were performed exclusively in the training set using stratified 5-fold cross-validation. Final performance estimates were obtained on the held-out test set to provide an unbiased assessment of generalization.

Model discrimination and classification performance were evaluated using the area under the ROC curve (AUC), accuracy, sensitivity, and specificity. ROC curves and AUC were computed using probabilistic outputs (predicted probabilities) rather than class labels to ensure valid discrimination assessment. In case of class imbalance, strategies such as class weighting and threshold evaluation were considered and reported when used.

To explore heterogeneity in patterns of healthcare utilization and related attributes, k-means clustering was applied to identify groups of individuals with similar profiles. Clustering was used as an exploratory tool to summarize multidimensional patterns rather than to infer causal relationships. Variables included in clustering were selected to reflect access-related characteristics; post-access outcomes were excluded, as the goal was to describe access profiles.

To examine contextual variability, a multilevel logistic regression model with a random intercept for community was fitted for the outcome of healthcare access. Between-community heterogeneity was summarized using the community-level variance component and the intraclass correlation coefficient (ICC) based on the latent-variable approximation for logistic mixed models.

Principal Component Analysis (PCA) was used to construct a Social Vulnerability Index (SVI) from socioeconomic and structural indicators (household income, education, housing type, employment status, transport mode to services/urban area, and race/color). PC1 was used as the synthetic vulnerability dimension and rescaled to a 0–100 score, with higher values indicating lower socioeconomic status. The SVI was used to describe access gradients across vulnerability strata and to explore associations between community-level mean SVI and access rates.

The study followed the ethical principles established in Resolution No. 466/2012 of the Brazilian National Health Council, ensuring autonomy, confidentiality, and the right to withdraw at any stage. All participants signed an informed consent form prior to participation. The project was approved by the Research Ethics Committee of the State University of Pará (UEPA), under approval number 4.915.684, and adhered to the principles of the Declaration of Helsinki. Data collection was conducted from April 10, 2024, to September 17, 2025.

The operational definition of healthcare access represents self-reported realized access among participants who perceived a need for care. It may conflate perceived need, demand, care-seeking, and receipt of services and does not provide a comprehensive measure of availability, affordability, acceptability, timeliness, continuity, or appropriateness of care. Consequently, the findings should not be interpreted as measuring the entire construct of healthcare access.

## Results

Table 1 summarizes the sociodemographic, territorial, household, and healthcare access characteristics of the 512 participants included in the study. The mean age was 40.6 ± 16.6 years, and the average number of completed years of schooling was 10.3 ± 4.5 years. Most participants resided in the communities of Tiningu (30.7%), Murumurutuba (21.3%), and Murumuru (20.5%).

**Table 1:** Sociodemographic, territorial, housing, and infrastructure characteristics of the study population from quilombola communities in the Brazilian Amazon (n = 512).

Characteristic	Category	n (%)
Total participants		512 (100.0)
Age (years)	Mean ± SD	40.6 ± 16.6
	Missing	4 (0.8)
Completed years of schooling	Mean ± SD	10.3 ± 4.5
	Missing	96 (18.8)
Community of residence	Arapemã	7 (1.4)
	Bom Jardim	62 (12.1)
	Ituqui	2 (0.4)
	João Pereira	12 (2.3)
	Murumuru	105 (20.5)
	Murumurutuba	109 (21.3)
	Pérola do Maicá	30 (5.9)
	Saracura	28 (5.5)
	Tiningu	157 (30.7)
Sex	Female	314 (61.3)
	Male	196 (38.3)
	Missing	2 (0.4)
Race/color	Black	423 (82.6)
	Brown	62 (12.1)
	White	11 (2.1)
	Indigenous	6 (1.2)
	Yellow/Asian	2 (0.4)
	Does not know	3 (0.6)
	Did not answer	2 (0.4)
	Missing	3 (0.6)
Marital status	Lives alone	150 (29.3)
	Lives with partner/family	337 (65.8)
	Did not answer	24 (4.7)
	Missing	1 (0.2)
Education level	Illiterate	7 (1.4)
	Elementary education	71 (13.9)
	Secondary education	153 (29.9)
	Higher education (incomplete/complete)	209 (40.8)
	Missing	72 (14.1)
Monthly household income	< R$ 250.00	42 (8.2)
	R$ 250.00–R$ 500.00	77 (15.0)
	R$ 501.00–R$ 1,500.00	212 (41.4)
	R$ 1,501.00–R$ 2,500.00	84 (16.4)
	R$ 2,501.00–R$ 4,000.00	36 (7.0)
	> R$ 4,000.00	17 (3.3)
	Does not know	23 (4.5)
	Missing	21 (4.1)
Currently working	Yes	120 (23.4)
	No	241 (47.1)
	Missing	151 (29.5)
Housing type	Masonry/brick	393 (76.8)
	Straw	6 (1.2)
	Mud/clay	6 (1.2)
	Wood	96 (18.8)
	Stilt house	4 (0.8)
	Other	5 (1.0)
	Missing	2 (0.4)
Drinking water source	River	18 (3.5)
	Well	135 (26.4)
	Microsystem/community supply	278 (54.3)
	Mineral water	19 (3.7)
	Other	49 (9.6)
	Missing	13 (2.5)
Household water treatment	Filtration	150 (29.3)
	Boiling	6 (1.2)
	Chlorination	163 (31.8)
	Other	22 (4.3)
	No treatment	160 (31.2)
	Missing	11 (2.1)
Sewage/feces and urine disposal	Septic tank	336 (65.6)
	Rudimentary pit/black pit	80 (15.6)
	Open defecation/open air	26 (5.1)
	Sewage system	45 (8.8)
	Other	11 (2.1)
	Missing	14 (2.7)
Bathroom inside household	Yes	398 (77.7)
	No	110 (21.5)
	Missing	4 (0.8)
Main access route to community	River	68 (13.3)
	Paved road	35 (6.8)
	Unpaved/dirt road	401 (78.3)
	Air	3 (0.6)
	Missing	5 (1.0)
Transport to health services/urban area	Boat	42 (8.2)
	Bus	344 (67.2)
	Truck/pickup	14 (2.7)
	Own transport	83 (16.2)
	Other	22 (4.3)
	Missing	7 (1.4)
Transport regularity	Daily	271 (52.9)
	Weekly	87 (17.0)
	Biweekly	21 (4.1)
	Monthly	113 (22.1)
	Other	8 (1.6)
	Missing	12 (2.3)
Electricity in community/household	Yes	241 (47.1)
	No	8 (1.6)
	Other/source reported	254 (49.6)
	Missing	9 (1.8)
Internet access	Yes	455 (88.9)
	No	38 (7.4)
	Alternative communication	13 (2.5)
	Missing	6 (1.2)

The study population was predominantly female (61.3%) and self-identified as Black (82.6%). Most participants lived with a partner and/or family members (65.8%). Regarding education, higher education (incomplete or complete) was the most frequent category (40.8%), followed by secondary education (29.9%). Household income was concentrated in the range between R$ 501.00 and R$ 1,500.00 (41.4%), while nearly half of the participants reported not currently working (47.1%).

Concerning housing conditions, most households were constructed of masonry/brick (76.8%), had an indoor bathroom (77.7%), and reported sewage disposal through septic tanks (65.6%). The main source of drinking water was microsystem/community supply (54.3%), although a substantial proportion of households reported no water treatment (31.2%) or chlorination as the primary treatment method (31.8%).

Territorial accessibility was predominantly dependent on unpaved/dirt roads (78.3%), and bus transportation was the main means of travel to health services or urban areas (67.2%). More than half of the participants reported daily transport availability (52.9%). Regarding infrastructure, internet access was widely reported (88.9%), whereas electricity conditions varied, with 47.1% reporting regular electricity access and 49.6% reporting alternative or locally reported energy sources.

**Fig 1:**
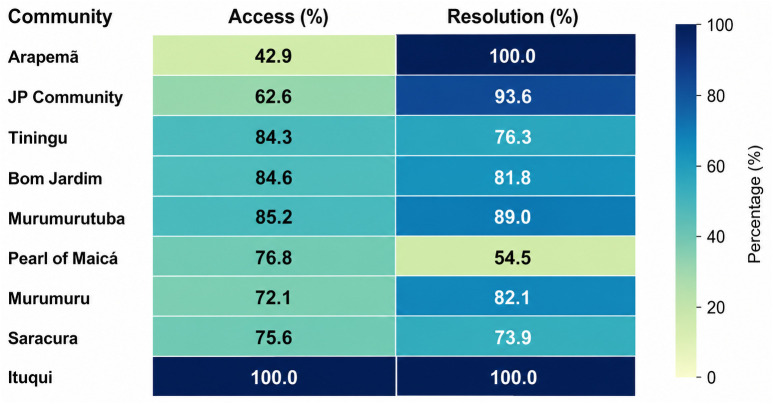
Heatmap of Access and Problem Resolution by Community.

Access to healthcare services showed substantial heterogeneity across communities (Fig 1). Tiningu, Murumuru, and Murumurutuba comprised the largest share of observations and displayed access rates of 64.3% (n = 154), 72.1% (n = 104), and 68.5% (n = 108), respectively. When resolution was assessed only among participants who reported accessing care and provided a valid response, resolution rates were highest in Murumurutuba (93.0%; 71 valid responses), followed by Murumuru (88.7%; 71 valid responses) and Tiningu (81.3%; 96 valid responses). Communities with smaller sample sizes should be interpreted cautiously due to wider sampling variability.

Table 2 summarizes CART performance in the held-out test set (20% of the sample; n = 103). To address concerns regarding inconsistent AUC reporting and improve reproducibility, model performance is presented using two complementary estimates: (i) mean AUC from 5-fold cross-validation (CV) within the training set, and (ii) final performance on a held-out test set (20% split; stratified). Overall discrimination was modest, and models showed high sensitivity with low specificity at the default 0.50 probability threshold, reflecting the high prevalence of “access” in the sample and the difficulty in correctly identifying the minority class (“no access”) without threshold optimization.

**Table 2:** Performance of the Decision Tree (CART) Model for Healthcare Access (P2).

Metric	No Access	With Access	Weighted Average
Precision	0.294	0.667	0.547
Sensitivity (Recall)	0.303	0.657	0.544
F1-score	0.299	0.662	0.545
Support (n)	33	70	103

Table 3 compares five classification algorithms using mean AUC from 5-fold cross-validation in the training set and performance on the held-out test set. Overall, discrimination was low to moderate (test AUC range: 0.482–0.586). Gradient Boosting achieved the highest test AUC (0.586), while Random Forest showed the highest sensitivity (0.986) but very low specificity (0.061) at the default threshold.

**Table 3:** Performance of predictive models for healthcare access prediction.

Model	AUC (CV – training)	AUC (Test)	Accuracy (Test)	Sensitivity (Test)	Specificity (Test)
Gradient Boosting	0.585	0.586	0.660	0.929	0.091
Random Forest	0.572	0.579	0.689	0.986	0.061
Logistic (Lasso)	0.600	0.557	0.631	0.829	0.212
Logistic (Ridge)	0.595	0.543	0.650	0.857	0.212
Decision Tree (CART)	0.577	0.482	0.544	0.657	0.303

Cluster analysis identified three distinct user profiles, reflecting heterogeneous patterns of healthcare access and problem resolution across the studied communities. Cluster 1 presented the lowest levels of access and resolution, predominantly involving users who sought care outside their communities. Cluster 2 showed intermediate indicators and greater participation of nursing technicians and assistants in care delivery. In contrast, Cluster 3 demonstrated the most favorable outcomes, with higher levels of access and problem resolution, mainly among users assisted within their own communities or at primary healthcare units. These findings suggest the existence of distinct territorial and organizational contexts influencing healthcare utilization.

Penalized regression models (Table 4) identified community-based care and support from healthcare professionals as important positive predictors of healthcare access, particularly care provided within the community and medical assistance for acute conditions. Conversely, receiving care outside the municipality and lower educational attainment were negatively associated with access. These variables were subsequently incorporated into the hierarchical and Gini importance analyses.

**Table 4:** Penalized Logistic Regression (LASSO and RIDGE): main variables associated with healthcare access.

Variable	Coefficient (LASSO)	Coefficient (RIDGE)
P5 – Care provided within the community (1 = Yes)	+0.84	+0.79
P3 – Medical professional	+0.62	+0.57
P4 – Reason: acute illness	+0.54	+0.51
P6 – Care outside the municipality	−0.63	−0.59
P3 – Community health worker	+0.46	+0.41
Low educational level	−0.40	−0.37

Table 5 presents the most influential features in the Random Forest model after one-hot encoding. The highest contributions were observed for age and sex, followed by household/structural indicators (e.g., water treatment, electricity supply) and mobility-related variables (transport mode/cost), along with selected community indicators. These results suggest that access is more strongly patterned by demographic, household, and territorial constraints than by post-care perceptions.

**Table 5:** Variable importance according to the Random Forest Model.

Rank	Variable (feature)	Importance (Gini)
1	Age (years)	0.0520
2	Sex: female	0.0232
3	Sex: male	0.0230
4	Kinship relation: 3,9	0.0170
5	Religion: Catholic	0.0148
6	Household water treatment: filtration	0.0146
7	Community: Tiningu	0.0142
8	Self-rated health (category)	0.0129
9	Currently working: not	0.0123
10	Transport to services/urban area (category)	0.0116
11	Electricity supply: yes	0.0114
12	Race/Color: brown	0.0110
13	Self-rated health (category)	0.0110
14	Transportation cost: R$12,00	0.0110
15	Education: elementary education	0.0109

Table 6 presents the fixed-effect estimates from the multilevel logistic regression model for healthcare access, adjusted for sex, age, education, and household income, with a random intercept for community. Female participants showed higher odds of reporting access compared with males (OR = 2.03; approximate 95% interval: 1.56–2.65). Age was positively associated with access, although with a small effect size per year (OR = 1.007; 1.002–1.011). Education and income categories displayed heterogeneous associations relative to the reference groups (no formal schooling and household income < R$ 250.00), with wide and overlapping uncertainty intervals for several levels, suggesting limited precision for some contrasts. The “does not know” income category showed higher odds of access relative to the lowest-income reference group (OR = 2.72; 0.95–7.79), a pattern that may reflect systematic differences in non-response and should be interpreted cautiously.

**Table 6:** Fixed effects from the multilevel logistic regression model for healthcare access.

Predictor	Coef (log-odds)	SD	OR	OR (approx. 95% interval)
Intercept	0.043	0.102	1.044	0.856–1.274
Sex: Female (vs Male)	0.710	0.134	2.033	1.562–2.646
Sex: Other/not reported (vs Male)	1.045	1.612	2.844	0.121–66.980
Education: Incomplete primary (vs No formal schooling)	0.319	0.278	1.375	0.798–2.372
Education: Complete primary / incomplete secondary (vs No formal schooling)	0.075	0.186	1.078	0.748–1.552
Education: Complete secondary (vs No formal schooling)	−0.116	0.154	0.890	0.659–1.203
Education: Higher education (vs No formal schooling)	−0.194	0.285	0.824	0.471–1.441
Income: R$ 250.00–R$ 500.00 (vs < R$ 250.00)	−0.185	0.243	0.831	0.517–1.338
Income: R$ 501.00–R$ 1,500.00 (vs < R$ 250.00)	0.051	0.154	1.052	0.778–1.423
Income: R$ 1,501.00–R$ 2,500.00 (vs < R$ 250.00)	0.112	0.243	1.119	0.695–1.801
Income: R$ 2,501.00–R$ 4,000.00 (vs < R$ 250.00)	0.038	0.371	1.039	0.502–2.149
Income: > R$ 4,000.00 (vs < R$ 250.00)	0.740	0.558	2.096	0.703–6.254
Income: Does not know (vs < R$ 250.00)	1.000	0.537	2.718	0.948–7.793
Age (per 1-year increase)	0.0068	0.0024	1.0068	1.002–1.011

As shown in Table 7, the estimated between-community variability in healthcare access was small after adjustment for individual covariates. This indicates that, in the fitted model, only a small proportion of the residual variation in access was attributable to differences between communities, while most variation remained at the individual level or was explained by measured covariates.

**Table 7:** Random-intercept variance and ICC (multilevel logistic model).

Component	Value
Random intercept SD (community)	0.201
Random intercept variance (community)	0.040
Approximate ICC	0.012

Table 8 shows that the PCA did not yield a single dominant dimension of vulnerability. PC1 explained 24.5% of the total variance, followed by PC2 (19.4%) and PC3 (16.2%), indicating that the socioeconomic and structural indicators capture a multidimensional vulnerability profile rather than a single underlying factor.

**Table 8:** Variance explained by Principal Components (PCA).

Component	Explained Variance (%)
PC1	24.5
PC2	19.4
PC3	16.2
PC4	15.2
PC5	12.5

The loadings in PC1 indicate that the vulnerability gradient was mainly driven by housing type and transport mode to services/urban area. In contrast, race/color showed a near-zero loading, suggesting minimal contribution to PC1 in this dataset (Table 9).

**Table 9:** Variable Weights in Principal Component 1.

Social determinant Variable	Weight (PC1)
Household income	−0.335
Education	−0.424
Housing type	0.562
Currently working	−0.349
Transport mode to services/urban area	−0.521
Race/Color	−0.003

At the individual level, access rates across SVI terciles showed limited separation (Table 10). At the community level (n ≥ 10), mean SVI was negatively correlated with access (r ≈ −0.53), suggesting a vulnerability gradient; however, estimates should be interpreted cautiously given the small number of communities.

**Table 10:** Access rates by terciles of the Social Vulnerability Index (SVI).

Vulnerability Group	% with Access
Low vulnerability	75.0
Medium vulnerability	69.7
High vulnerability	71.9

**Fig 2:**
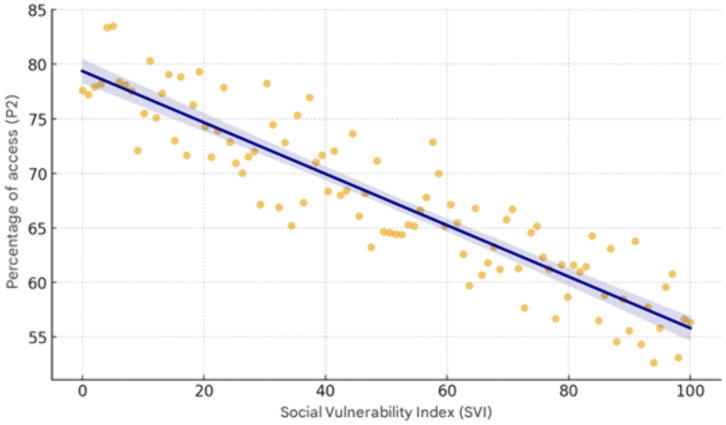
Correlation Between the Social Vulnerability Index (SVI) and the Percentage of Healthcare Access.

Fig 2 shows the relationship between the Social Vulnerability Index (SVI) and the percentage of healthcare access (P2) in the communities studied. Each point represents a community, while the blue line indicates the linear fit with a 95% confidence interval. At the community level (restricting to communities with n ≥ 10), mean SVI showed a negative correlation with access rates, consistent with a vulnerability gradient, though estimates should be interpreted cautiously due to the small number of communities meeting this threshold.

**Fig 3:**
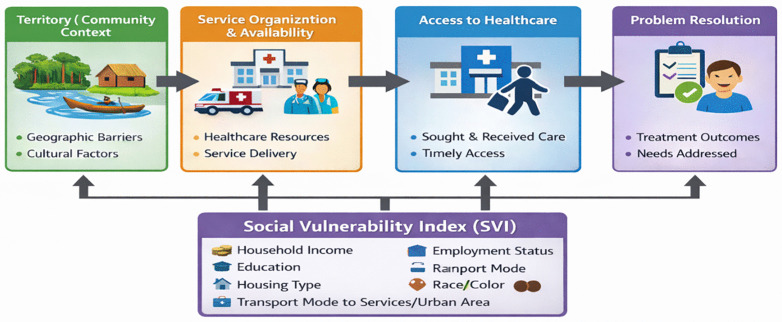
Conceptual Framework of Social Vulnerability and Healthcare Access in Quilombola Communities of the Brazilian Amazon.

This conceptual framework illustrates the hypothesized relationships between territorial context, service organization, healthcare access, and problem resolution in quilombola communities (Fig 3). The model is structured in four sequential domains:

(1) Territory/Community Context, including geographic barriers and cultural factors; (2) Service Organization & Availability, representing healthcare resources and service delivery; (3) Access to Healthcare, encompassing sought and received care as well as timely access; and (4) Problem Resolution, reflecting treatment outcomes and needs addressed. The SVI is modeled as a structural determinant influencing territorial conditions, healthcare organization, and access dynamics, thereby shaping the likelihood of problem resolution.

## Discussion

The results of this study demonstrate that access to healthcare services in the rural and riverside communities analyzed of western Pará remains deeply conditioned by social and territorial factors. Although the SUS is based on a universal model of care, the Amazonian territory imposes geographic and logistical barriers that undermine equity and continuity of care. Communities located farther from primary health units and with fewer permanent professionals showed the lowest access and resolution rates, suggesting that both physical space and the organization of service delivery remain structural determinants of health exclusion [6,21,22].

The machine learning analyses reinforced the multifactorial nature of healthcare access. Variables related to the type of professional, location of care, and reason for seeking assistance emerged as central predictors in the supervised models. These results align with previous studies conducted in areas with low service density, which highlight the decisive role of organized primary care and community health workers as facilitators of access. Thus, beyond the mere presence of healthcare facilities, the effectiveness of the system depends on the continuous and contextually grounded presence of qualified professionals [23,24].

The strong association between receiving care within the community and higher access rates underscores the importance of territorialized primary care strategies. In riverside and floodplain regions, traveling to urban centers entails not only financial cost but also the disruption of the bond between users and healthcare teams. Literature on primary care in remote areas consistently emphasizes that the regular presence of mobile or fluvial health teams, in coordination with local community health programs, represents one of the main mechanisms for mitigating territorial inequalities [25,26].

The results of the penalized models (Lasso and Ridge) converged in identifying the most influential variables, reinforcing the robustness of the findings. The positive association between care provided by physicians or nurses and the probability of access indicates that professional qualification and team comprehensiveness strengthen user trust and stimulate service utilization [27]. Conversely, low educational attainment and the need to travel outside the municipality emerged as significant barriers, highlighting the persistent role of social inequality and deficient infrastructure as determinants of non-access.

Cluster analysis identified three distinct profiles of healthcare utilization. The first group, characterized by low access and resolution rates, included individuals from more remote communities with limited fixed services. The second group represented an intermediate profile, with moderate access and a predominance of care provided by nursing technicians and assistants. The third cluster grouped users with greater integration into the primary care network and higher satisfaction and resolution indicators. This segmentation illustrates the multiple realities of the Amazonian health system, where significant advances coexist with persistent structural gaps.

The application of ensemble models, such as *Random Forest* and *Gradient Boosting*, confirmed that the pattern of access cannot be explained solely by individual variables, but rather results from a complex interaction of social, professional, and territorial dimensions. The substantial weight of variables related to community of residence and type of service supports the hypothesis that local service structure directly influences utilization. This finding converges with studies on the territorialization of SUS, which emphasize that community context, more than individual profile, can define the probability of access.

The multilevel model confirmed that most of the observed variance is concentrated in individual and organizational factors, with a low intraclass correlation coefficient across communities. This does not indicate the absence of territorial inequality but rather that, after statistical adjustment, structural disparities manifest primarily through mediating variables, such as service type and professional profile. In practice, this suggests that territory functions as an indirect determinant, shaping opportunities and conditions of access through the organizational structure of the health system [28–30].

The construction of the Social Vulnerability Index (SVI) made it possible to synthesize socioeconomic dimensions into a sensitive and interpretable indicator. The inverse gradient between vulnerability and access observed across SVI terciles reinforces the well-known paradox of inequity: those in the poorest social conditions are precisely those who access the system the least. This pattern, widely documented in different Brazilian contexts, becomes more severe in the Amazon, where physical barriers and infrastructural limitations amplify the effects of poverty on healthcare [31].

By integrating machine learning, multilevel modeling, and principal component analysis, this study demonstrated the potential of computational approaches to reveal complex health inequalities. Unlike traditional linear models, the algorithms used were capable of capturing non-linear interactions and identifying high-impact variables without assuming fixed causal relationships. This methodological flexibility is particularly valuable in heterogeneous territories, where variable behavior may differ significantly across groups and contexts [32,33].

The findings highlight the need for public policies that incorporate the territorial dimension as a structuring axis of health planning. Expanding fluvial primary care teams, strengthening community health education, and investing in technological strategies for remote monitoring represent viable pathways to reduce inequalities and enhance system equity [34–36]. By evidencing how social and geographic factors intertwine in the production of inequities, this study reaffirms that guaranteeing access to healthcare in the Amazon requires not only infrastructure investment but also innovative analytical approaches capable of translating territorial complexity into actionable insights for public management [37–41].

This study has limitations. First, its cross-sectional design precludes establishing temporal ordering or causal relationships between socioeconomic conditions, territorial characteristics, service organization, self-reported healthcare access, and problem resolution. The reported estimates should therefore be interpreted as associations and exploratory patterns rather than causal effects.

Second, healthcare access was operationalized as self-reported realized access, defined as receipt of care following a perceived need during the previous 12 months. This measure captures only one stage of the access process and does not distinguish all dimensions of need, demand, care-seeking, availability, affordability, acceptability, timeliness, continuity, and appropriateness. It may also exclude unrecognized or unexpressed healthcare needs.

Third, need for care, receipt of services, and problem resolution were based on self-report and may be affected by recall bias, differences in interpretation, and social-desirability bias. These factors may have produced outcome misclassification. If reporting varied according to socioeconomic characteristics, community of residence, prior experiences with services, or interviewer interaction, the magnitude and direction of the observed associations may have been affected.

Fourth, recruitment was community-based and non-probabilistic and prioritized residents who were present and available during data collection. This approach may have overrepresented participants with greater availability, stronger community engagement, or different patterns of healthcare use and may have underrepresented workers, mobile residents, individuals living in less accessible areas, or those unwilling to participate. The sample-size calculation therefore does not guarantee population representativeness.

Fifth, community-level analyses were based on only nine communities with markedly unequal sample sizes. Some communities had very few participants, and analyses restricted to communities with at least 10 observations involved an even smaller number of aggregate units. Consequently, ecological correlations, variance components, and ICC estimates may be unstable and sensitive to individual communities. Community-level associations cannot be directly attributed to individuals and should be regarded as descriptive and hypothesis-generating.

Sixth, the predictive models showed limited discrimination in the held-out test set, indicating restricted generalizability for individual-level prediction. Variable-importance measures describe the fitted models and should not be interpreted as causal effects. External validation in larger, probabilistically selected, and geographically diverse samples is required.

The findings derive from quilombola rural and riverside communities in a specific region of western Pará and may not be generalizable to other quilombola populations, urban communities, Indigenous populations, or Amazonian territories with different service networks, mobility patterns, and sociocultural characteristics.

## Conclusion

In the communities investigated, self-reported healthcare access varied across social, organizational, and territorial characteristics. Differences according to community of residence, transport conditions, service location, and socioeconomic vulnerability suggest that opportunities to obtain care are unevenly distributed within this Amazonian setting. Because of the cross-sectional design, these findings represent associations and exploratory patterns and do not establish temporal or causal relationships.

The predictive models showed limited discrimination in the independent test set, indicating that the variables available in the study were insufficient to accurately distinguish participants with and without reported access. Thus, the main contribution of the machine-learning analyses lies in exploratory pattern recognition rather than individual prediction or causal explanation. Community-level findings should likewise be interpreted descriptively because they were based on a small number of communities and cannot be directly transferred to individual-level relationships or to other Amazonian populations.

The results support the need for locally adapted strategies addressing transportation, continuity of primary care, referral pathways, and digital and territorial infrastructure. Future longitudinal and multisite studies should examine whether these factors prospectively influence healthcare access and should employ broader multidimensional measures of unmet need and service accessibility.

## Supporting information

S1 file (XLSX)

## Abbreviations

AUC: Area Under the Curve
CART: Classification and Regression Tree
CI: Confidence Interval
ICC: Intraclass Correlation Coefficient
ML: Machine Learning
PCA: Principal Component Analysis
PC1: Principal Component 1
RF: Random Forest
ROC: Receiver Operating Characteristic
SVI: Social Vulnerability Index
SUS: Sistema Único de Saúde (Brazil’s Unified Health System)
UEPA: Universidade do Estado do Pará (State University of Pará)
UFOPA: Universidade Federal do Oeste do Pará (Federal University of Western Pará)
