# Supplementary material for: Healthcare access inequalities in Quilombola rural and riverside communities in the Brazilian Amazon: Associations with social, organizational, and territorial factors
Source: PLoS One. 2026 Aug 10;21(8):e0355489. doi: 10.1371/journal.pone.0355489 (PMC13456258; doi:10.1371/journal.pone.0355489)
Supplement: Supplementary file 1 [file pone.0355489.s002.pdf]

LETTER OF REPLY

Data Availability Statement

R: There are no ethical or legal restrictions preventing the sharing of the anonymized dataset used in this study. The dataset has been fully anonymized prior to public release and does not contain any direct identifiers or information that could reasonably lead to participant identification. The study was approved by the Research Ethics Committee of the State University of Pará (UEPA), approval number 4.915.684, and all procedures complied with Brazilian ethical regulations and the Declaration of Helsinki.

The minimum dataset required to reproduce the analyses reported in this manuscript, together with the corresponding codebook describing all variables and coding procedures, is publicly available and may be accessed through the following DOI: https://doi.org/10.1371/journal.pntd.0014078.s001

This repository contains the anonymized dataset supporting the findings of the study, including sociodemographic, environmental, territorial, and health-related variables used in the statistical and machine-learning analyses. Therefore, there are no restrictions on data access, and no data access committee is required. All interested researchers may freely access the dataset through the DOI provided above.

Funding Statement

R: This study received no specific funding from any public, commercial, or not-for-profit funding agency. Although some co-authors are undergraduate medical students affiliated with Metropolitan College of Pará – FAMETRO, Santarém, Pará, Brasil and University of Amazonia – UNAMA, Santarém, Pará, Brasil, these institutions did not provide financial support, salaries, research materials, infrastructure, or any other resources for this study. The research was conducted within the framework of the State University of Pará (UEPA), a public university. The participation of students from FAMETRO and UNAMA occurred on a voluntary basis and was unrelated to any institutional sponsorship, employment relationship, or financial support. Therefore, no commercial entity had any role in the study design, data collection, data analysis, decision to publish, or preparation of the manuscript.

Competing Interests Statement

The authors declare that they have no competing interests. The editorial office identified affiliations with Metropolitan College of Pará – FAMETRO, Santarém, Pará, Brasil and University of Amazonia – UNAMA, Santarém, Pará, Brasil as potential commercial affiliations. However, the authors affiliated with these institutions are undergraduate students and are not employees, consultants, shareholders, or representatives of these organizations. These institutions did not provide funding, salaries, materials, infrastructure, or any form of support for this research. The participation of these authors was entirely voluntary and independent of their academic affiliations. This does not alter our adherence to PLOS ONE policies on sharing data and materials.

REVIEWER 1

1. Misalignment between the study objective and study design. The manuscript aims to “identify determinants” of healthcare access. However, given the cross-sectional design, this wording overstates the analytical capacity of the study. Cross-sectional studies do not establish temporality and are not suitable for identifying determinants in a causal sense. The objective should be revised to reflect the exploratory and associative nature of the analysis.

Response: We thank the reviewer for this observation. We clarify that we consider healthcare access as a multidimensional and territorially conditioned phenomenon, a concept derived from the Social Determinants of Health framework widely used in health research, particularly in the Amazon region and strongly linked to the healthcare system adopted in Brazil. To improve methodological precision and avoid causal interpretations, we revised the study objective by replacing the verb “identify” with “investigate.” The objective now reads: “The present study aimed to investigate social and territorial determinants of healthcare access in the Brazilian Amazon.”

2. Conceptual weakness of the primary outcome (“healthcare access”). The outcome is based on self-reported receipt of care when needed, which may conflate need, demand, and access and is subject to recall and reporting biases. A more robust conceptual framework is needed to support this operationalization and avoid overinterpretation.

Response: To provide a more robust conceptual framework regarding the theoretical conception of access adopted in this study, we revised the manuscript and added the following text to the Methods section (lines 151–159): “This approach was adopted considering the organization of health services in the Amazon region and the geographical and organizational characteristics of the communities studied, which are related to the effective provision of care in the context of the healthcare system. In this sense, access no longer depends exclusively on spontaneous demand or individual perception of need, but rather on the organization and interrelation of services, referral and counter-referral processes, the implementation of active search strategies, the presence of a multidisciplinary team in the territory, and the ability to travel to access or continue using the healthcare network.”

3. Insufficient discussion of potential biases. Major sources of bias are not adequately addressed, including selection bias (community-based recruitment) and information bias (self-reported measures).

Response: We thank the reviewer for this observation. We expanded the discussion of methodological biases present in the study, with particular emphasis on information bias. In addition, we detailed the possible implications of recall bias, social desirability bias, and community-based recruitment. The following sections were revised: “Second, several variables were based on self-reported information, which may be subject to recall bias, leading to the oversimplification or underreporting of older events, an overemphasis on more memorable experiences at the expense of routine healthcare visits, and distorted perceptions resulting from the fragmented care context experienced in the region. In addition, they may be influenced by social desirability bias, stemming from

embarrassment when reporting information, possible influence by interviewers, and the historical context of vulnerability of the studied population, generating fear of negative consequences for the community or personal relationships with the health service."

"The study was conducted in quilombola communities within a specific region of the Brazilian Amazon, whose territorial and logistical characteristics (population dispersion, seasonal and precarious displacement, high-cost transportation, and geographic isolation) increase the complexity of fieldwork and hinder population recruitment..."

4. Absence of a descriptive table of sample characteristics. The Results section does not present a comprehensive table describing the study population. This limits interpretability and the assessment of external validity. Inclusion is recommended according to the STROBE Statement.

Response: A table describing the sociodemographic characteristics of the analyzed sample was included.

5. Potential overinterpretation of community-level results. Results based on aggregated data should be interpreted cautiously, considering the limited number of communities and the risk of ecological inference.

Response: We thank the reviewer for this observation. We revised the Discussion and Conclusion sections to provide a more cautious interpretation of the findings at the community level, correcting passages that could suggest extrapolation or generalization beyond the inferential scope of the study. We also reinforced the study limitations to make clear the need for caution when interpreting and generalizing the findings.

REVIEWER 2

1. It is necessary to include a map contextualizing the location of the communities used in the study, to provide a better understanding of how isolated these communities are, since not all readers are familiar with the Amazonian reality.

Response: A map was included to contextualize the analyzed communities.

2. I would recommend changing the title to: "Healthcare Access Inequalities in Quilombola Rural and Riverside Communities in the Brazilian Amazon: Social, Organizational, and Territorial Determinants." With the original title, it appears that three different community types (quilombola, rural, and riverside) are being analyzed separately, whereas the methodology makes it clear that these characteristics refer to the same communities.

Response: We thank the reviewer for the suggestion. The title was revised and changed to: "Healthcare Access Inequalities in Quilombola Rural and Riverside Communities in the Brazilian Amazon: Social, Organizational, and Territorial Determinants."

3. Line 125: Remove "either".

Response: Removed.

4. Lines 174–176: Information repeated from lines 161–164.

Response: Verified and redundant information was removed.

5. Line 193: Lasso and Ridge (only the first letter capitalized).

Response: Verified and corrected.

6. Lines 202–203: Repetition of information from lines 200–201.

Response: Verified and redundant information was removed.

7. Line 205: Replace "when class imbalance was present" with "In cases of class imbalance".

Response: Verified and replaced.

8. Lines 211–212: Replace with "Variables included in clustering were selected to reflect access-related characteristics; post-access outcomes were excluded, as the goal was to describe access profiles."

Response: Verified and replaced.

9. Line 215: Replace with "was adapted for the healthcare access outcome".

Response: Verified and replaced**.**

10. Line 223: Replace with "lower socioeconomic status".

Response: Verified and replaced.

11. Line 234: "Among the 517 records in the database, 512 participants..." This information already appears in the Methods section.

Response: Verified and lines 234–235 were removed.

12. Line 277: Table 3 is unnecessary because the information is repeated in the text.

Response: Verified and Table 3 was removed.

13. Line 288: Table 4 is unnecessary because all information is repeated in the text.

Response: We chose to retain the table and revise the preceding text into an interpretative synthesis of the findings, avoiding repetition of coefficients already presented in the table and reducing redundancy.

14. Line 281: Change to "Lasso" and "Ridge".

Response: Verified and corrected.

15. Line 322: Table 7 is unnecessary because all information is repeated in the text.

Response: We chose to retain the table and removed the sentence "The community-level random-intercept standard deviation was 0.201 (variance = 0.040), corresponding to an approximate ICC of 0.012," maintaining the interpretation of the findings while avoiding duplication.

16. Line 336: Table 9 is unnecessary because all information is repeated in the text.

Response: Numerical values and the phrase “with additional contributions from education (−0.424), employment status (−0.349), and household income (−0.335)” were removed from the text preceding Table 9, maintaining the interpretation while avoiding repetition.

17. Line 393: Lasso, only the first letter capitalized.

Line 415: Correct to “context”.

Response: Verified and corrected.

18. Line 429: Replace “assumes more dramatic contours” with “becomes more severe”.

Response: Verified and replaced.

19. Question: I would like to know why telehealth services were not discussed in greater depth. There are many articles on this topic, including in the Amazon region itself. It is mentioned only briefly. In my opinion, this topic should be addressed given its relevance.

Response: We thank the reviewer for this observation and recognize the relevance of telehealth as a strategy to expand healthcare access, particularly in the Brazilian Amazon. We added a paragraph addressing this issue. However, we chose not to discuss it in greater depth because telehealth/telemedicine services were not available in the communities investigated during the study period and were not identified by participants as part of their healthcare access experience. Therefore, although the topic is relevant to the broader discussion, it was not part of the investigation or analytical framework of the present study and was mentioned only as a potential facilitator of healthcare access.
