# Supplementary material for: Healthcare access inequalities in Quilombola rural and riverside communities in the Brazilian Amazon: Associations with social, organizational, and territorial factors
Source: PLoS One. 2026 Aug 10;21(8):e0355489. doi: 10.1371/journal.pone.0355489 (PMC13456258; doi:10.1371/journal.pone.0355489)
Supplement: Supplementary file 2 [file pone.0355489.s003.docx]

RESPONSE TO THE EDITOR AND REVIEWERS

Manuscript title: Healthcare Access Inequalities in Quilombola Rural and Riverside Communities in the Brazilian Amazon: Associations with Social, Organizational, and Territorial Factors

Dear Editor and Reviewers,

We sincerely thank the Editor and the Reviewers for the careful re-evaluation of our manuscript and for the detailed and constructive recommendations. We appreciate the recognition that the previous revision improved the methodological description, contextualization, and presentation of the findings.

We agree with the Editor that some of our previous responses described the changes made without sufficiently explaining the scientific rationale underlying those changes. We therefore conducted a comprehensive revision of both the manuscript and the response letter. In the present response, each item specifies: (1) what was changed; (2) where the change can be found; and (3) how the modification addresses the methodological, conceptual, or editorial concern raised.

All changes have been highlighted in the revised manuscript.

ADDITIONAL COMMENTS FROM THE EDITOR

**Editor comment 1:**

Please ensure that all reviewer comments are addressed in full. In several comments, the response letter states that a change was made, but the revision does not always fully resolve the underlying concern. In particular, the conceptual definition of healthcare access should be strengthened while explicitly acknowledging the limitations of the operational definition, and the limitations of the cross-sectional design should be discussed more explicitly regarding temporality and causality.

**Response:**

We thank the Editor for this important observation. We agree that our previous response did not fully distinguish the broad theoretical construct of healthcare access from the narrower operational measure used in this study.

Three substantive changes were made.

First, we reformulated the objective so that it no longer suggests causal identification. The revised objective now reads:

“The present study aimed to examine individual, organizational, and territorial factors associated with self-reported healthcare access among quilombola rural and riverside communities in Santarém, Brazilian Amazon, using conventional statistical and machine-learning approaches.”

This wording appears in the Abstract, lines 22–26, and at the end of the Introduction, lines 108–113. The terms “examine” and “associated with” were selected because the cross-sectional design permits the examination of associations and exploratory patterns but does not establish temporal ordering or causal determination.

Second, we substantially revised the operational definition of the primary outcome in the Methods, lines 162–179. Healthcare access is now explicitly defined as self-reported realized access, namely, receipt of healthcare after the participant perceived a need during the previous 12 months. We also state that this measure captures only one stage of the access process and does not represent a comprehensive assessment of healthcare accessibility. The manuscript now explicitly acknowledges that the measure does not fully distinguish health need, perceived need, demand, care-seeking, service availability, utilization, timeliness, continuity, or adequacy of care.

Third, we expanded the limitations in the Discussion, lines 485–527, to explain that the cross-sectional design precludes establishing whether the social, territorial, and organizational characteristics preceded or resulted from the reported access experience. We therefore state that the findings must be interpreted as associations and hypothesis-generating patterns rather than causal effects.

These revisions address the Editor’s concern by aligning the conceptual interpretation of the outcome and the reported findings with the actual measurement strategy and the inferential limits of the study design.

**Editor comment 2**

The responses should explain why, and not only what. Each response should clearly explain what changes were made, where the changes can be found, and how they address the reviewer’s concern.

**Response**

We agree and have completely restructured the response letter. Each response now includes: the specific textual, methodological, or analytical modification; the section and current line numbers in which the modification appears; an explanation of why the modification resolves or mitigates the concern raised.

Responses that previously stated only “verified,” “corrected,” “removed,” or “revised” have been replaced with complete explanations. For minor grammatical corrections, we identify the revised passage and explain that the modification improves linguistic accuracy or removes redundancy without altering the substantive meaning. For methodological issues, we explicitly describe the inferential or conceptual implications of the revision.

**Editor comment 3**

When a reviewer’s recommendation is not followed, provide a scientific justification. The authors are not required to implement every suggestion, but retaining a table or text should be scientifically justified.

**Response**

We thank the Editor for this clarification. We have expanded the responses concerning Tables 4, 7, and 9, which were retained despite the Reviewer’s recommendations for removal.

Table 4 was retained because it reports the complete coefficients from the Lasso and Ridge models and enables readers to evaluate the direction, relative magnitude, and consistency of the associations across the two penalized approaches. The accompanying Results text was shortened so that it now provides only an interpretative synthesis rather than repeating all coefficients. The revised text and table are located in the Results, lines 326-339.

Table 7 was retained because reporting the community-level random-intercept standard deviation, variance, and approximate ICC is necessary for transparent evaluation of contextual heterogeneity. These parameters cannot be adequately evaluated from a narrative statement alone. Numerical duplication was removed from the preceding paragraph, which now focuses on substantive interpretation. The revised paragraph and table are located in the Results, lines 361-372.

Table 9 was retained because interpretation and reproducibility of a PCA-derived index require transparent reporting of component loadings. The loadings show how each socioeconomic and structural variable contributed to the first component and allow readers to assess the substantive meaning of the Social Vulnerability Index. Numerical values were removed from the narrative and retained only in the table. The revised material appears in the Results, lines 375-383.

These decisions preserve the information necessary for analytical transparency while eliminating textual duplication.

**Editor comment 4**

Please verify consistency throughout the manuscript. In particular, revisions to the study objective should be reflected consistently in the Abstract, Introduction, manuscript metadata, cover letter, and response letter.

**Response**

We performed a systematic terminology review across the manuscript and accompanying submission documents.

The study objective has been standardized as:

“To examine individual, organizational, and territorial factors associated with self-reported healthcare access among quilombola rural and riverside communities in Santarém, Brazilian Amazon, using conventional statistical and machine-learning approaches.”

This wording appears in the Abstract, lines 22-26, and the Introduction, lines 108–113, and has also been incorporated into the revised cover letter, submission-system metadata, and response letter.

We also revised the study-design terminology. The study is now described in the Methods, lines 114–120, as a: “community-based cross-sectional survey”, rather than a population-based study. The same wording has been introduced in the Abstract.

Throughout the Results, Discussion, and Conclusion, causal formulations were revised, particularly when referring to the empirical findings. Terms such as “caused,” “determined,” “proved,” and “confirmed” were replaced, where appropriate, by “was associated with,” “showed a pattern,” “suggested,” or “was consistent with.”

The Conclusion, lines 528-536, now explicitly states that the findings represent associations and exploratory patterns and do not establish temporal or causal relationships.

The revised title was also aligned with this interpretation: “Healthcare Access Inequalities in Quilombola Rural and Riverside Communities in the Brazilian Amazon: Associations with Social, Organizational, and Territorial Factors.”

This terminology audit ensures consistency between the study objective, design, outcome definition, analytical strategy, and inferential claims.

**Editor comment 5**

Please carefully review the revised Data Availability Statement. If the dataset is now publicly available, ensure that the repository corresponds to the dataset used in the present study and that the statement is consistent with the submission system. If availability changed since the original submission, clearly explain the change.

**Response**

We thank the Editor for identifying the need to clarify the change in the Data Availability Statement. In the original submission, we adopted a conservative position regarding public data sharing because the study involved participants from small and geographically identifiable quilombola communities. We were particularly concerned about the possibility of indirect identification through combinations of territorial, demographic, and socioeconomic variables, even after the removal of direct personal identifiers.

Following the first review round, the dataset was reassessed and a minimum analytical dataset was prepared for public sharing. Direct identifiers were removed, and variables presenting a potential risk of indirect identification were reviewed, recoded, or organized in a manner intended to preserve participant confidentiality. The resulting dataset contains the observations and analytical variables required to support the findings reported in the manuscript, together with a codebook describing variable definitions, categories, and coding procedures.

The Data Availability Statement was revised in the manuscript, lines 575-584, and the same wording has been entered into the journal submission system. The revised statement reads:

“The anonymized dataset supporting the findings of this study is publicly available as Supporting Information. The dataset includes the variables analyzed in this study, comprising sociodemographic, environmental, territorial, and health-related information, as well as the corresponding codebook describing variable definitions, categories, and coding procedures used in the statistical and machine-learning analyses. All data were fully anonymized prior to sharing to ensure participant confidentiality and compliance with ethical standards for research involving vulnerable populations.”

The public file and its DOI were verified against the final analytical dataset before resubmission. Thus, the change from the initial statement reflects the preparation and verification of an anonymized minimum dataset, rather than a change in the ethical approval or an assumption that the original identifiable database could be shared without restriction.

Dataset DOI: <https://doi.org/10.1371/journal.pntd.0014078.s001>

**Editor comment 6**

Please ensure that the limitations explicitly discuss information bias associated with self-reported variables, selection bias related to community-based recruitment, and the limited number of communities included in contextual analyses and its implications for generalization.

**Response**

We agree and have substantially expanded the limitations section in the Discussion, lines 485-527. Regarding information bias, we now explain that healthcare need, receipt of care, and problem resolution were self-reported using a 12-month reference period. These measures may be affected by recall error, variation in individual interpretation, and social-desirability bias. We also state that these processes may have resulted in outcome misclassification and that, if reporting differed according to socioeconomic characteristics, community, previous service experiences, or interviewer interaction, the observed associations could have been attenuated or exaggerated.

Regarding selection bias, we now explicitly describe recruitment as community-based and non-probabilistic. Residents were included according to their presence, availability, and willingness to participate during fieldwork. We explain that this process may have overrepresented residents with greater availability or community engagement and underrepresented workers, mobile residents, people in less accessible areas, and individuals unwilling to participate. We further clarify that the sample-size calculation does not guarantee population representativeness.

Regarding contextual analyses, we now state that only nine communities were included and that their sample sizes were markedly unequal. Some communities contained very few participants, and analyses restricted to communities with at least 10 observations involved an even smaller number of aggregate units. Consequently, ecological correlations, variance components, and ICC estimates may be unstable and sensitive to individual communities. We explicitly state that community-level associations cannot be transferred directly to individual-level relationships and should be regarded as descriptive and hypothesis-generating.

We clarify that the findings may not be generalizable to other quilombola, urban, Indigenous, or Amazonian populations with different health-service networks, mobility conditions, and sociocultural characteristics.

REVIEWER 1

**Reviewer 1 - Comment 1**

Misalignment between the study objective and study design. The manuscript aims to “identify determinants” of healthcare access. However, given the cross-sectional design, this wording overstates the analytical capacity of the study.

**Response**

We thank the Reviewer and agree that the previous objective could be interpreted causally.

The objective was reformulated in the Abstract, lines 22–26, and the Introduction, lines 108–113, as follows:

“The present study aimed to examine individual, organizational, and territorial factors associated with self-reported healthcare access among quilombola rural and riverside communities in Santarém, Brazilian Amazon, using conventional statistical and machine-learning approaches.”

We replaced “identify determinants” with “examine factors associated with” because a cross-sectional study can estimate associations and describe patterns but cannot establish the temporal ordering required for causal interpretation.

The title, conclusion, cover letter, submission metadata, and response letter were also revised to use association-based terminology. In addition, the limitations and conclusion now explicitly state that the results do not establish temporal or causal relationships.

**Reviewer 1 - Comment 2**

Conceptual weakness of the primary outcome. The outcome is based on self-reported receipt of care when needed, which may conflate need, demand, and access and is subject to recall and reporting biases.

**Response**

We thank the Reviewer and agree that our previous description did not sufficiently distinguish the theoretical construct of healthcare access from its operational measurement in this study.

The primary outcome was redefined in the Methods, lines 162-179, as self-reported realized access: “The primary outcome was healthcare access operationalized as self-reported realized access, defined as having received healthcare when the participant perceived that care was needed during the previous 12 months.”

We now explicitly state that this measure captures only the successful receipt of care following a perceived need. It should not be interpreted as a comprehensive measure of healthcare accessibility and does not distinguish all stages between objective health need, perceived need, care-seeking, service availability, utilization, continuity, or adequacy.

We also explain that territorial mobility, transportation, primary-care organization, referral pathways, and health-system responsiveness were examined as contextual and organizational characteristics but were not assumed to be fully represented by the primary outcome.

The same limitation is reiterated in the Discussion, lines 490-502. This revision prevents the operational measure from being interpreted as encompassing the entire multidimensional construct of healthcare access.

**Reviewer 1 - Comment 3**

Major sources of bias are not adequately addressed, including selection bias and information bias.

**Response**

We agree and expanded both the Methods and Discussion.

In the Methods, lines 139-154, we now state that recruitment was community-based and non-probabilistic and included residents present and available during fieldwork. We explain that participation may have been associated with employment, mobility, health status, community engagement, or previous experiences with health services.

In the Discussion, lines 503-517, we describe how this process could overrepresent residents with greater availability or community involvement and underrepresent workers, mobile residents, those in less accessible locations, or people unwilling to participate.

We also explain that the self-reported measures may be affected by recall bias, differences in interpretation, and social-desirability bias. These processes may lead to outcome misclassification. If the reporting process varied according to social position, community, prior service experience, or interviewer interaction, the direction and magnitude of associations may also have been affected.

These revisions address not only the presence of possible biases but also their potential consequences for the estimated access rates and associations.

**Reviewer 1 - Comment 4**

The Results section does not present a comprehensive table describing the study population.

**Response**

We thank the Reviewer. A comprehensive descriptive table was included as Table 1, Results, lines 260-289.

The table presents the distribution of: age and years of schooling; community of residence; sex and race/color; marital status; education; household income and employment; housing type; water source and treatment; sanitation; internal bathroom availability; primary access route; transportation mode and regularity; electricity and internet access.

Missing observations are also reported by variable. This addition improves transparency regarding sample composition, missingness, territorial distribution, and the external validity of the findings, in accordance with STROBE recommendations.

**Reviewer 1 - Comment 5**

Community-level results may be overinterpreted. Aggregated findings should be interpreted cautiously because of the limited number of communities and the risk of ecological inference.

**Response**

We agree and revised the Results, Discussion, limitations, and Conclusion.

In the Results, lines 384-396, the relationship between mean SVI and community access rates is now described as exploratory. We explicitly state that the estimate should be interpreted cautiously because it was based only on communities with at least 10 observations.

In the limitations, lines 513-522, we explain that only nine communities were included, sample sizes were markedly unequal, and the ecological correlation involved an even smaller number of aggregate units. We state that these estimates may be unstable and influenced by individual communities and that community-level associations cannot be attributed directly to individuals.

In the Conclusion, lines 528-536, community-level findings are described as descriptive and not directly transferable to individual relationships or other Amazonian populations.

These changes reduce the risk of ecological overinterpretation and clearly define the contextual analyses as hypothesis-generating.

REVIEWER 2

**Reviewer 2 - Comment 1**

Include a map contextualizing the location and isolation of the communities.

**Response**

We thank the Reviewer. A map was included as Figure 1, Methods, lines 129–137.

The figure displays the spatial distribution of the nine quilombola rural and riverside communities relative to the urban center of Santarém. It complements the contextual description of road and river displacement and helps readers unfamiliar with the Amazonian setting understand the geographic dispersion of the communities.

**Reviewer 2 - Comment 2**

Change the title so that it is clear that quilombola, rural, and riverside refer to characteristics of the same communities rather than three separate populations.

**Response**

We thank the Reviewer and agree with the need to eliminate this ambiguity. We retained the sequence “quilombola rural and riverside communities,” indicating that rural and riverside are territorial characteristics of the quilombola communities investigated.

Following the Editor’s additional concern regarding causal terminology, we further refined the title to: “Healthcare Access Inequalities in Quilombola Rural and Riverside Communities in the Brazilian Amazon: Associations with Social, Organizational, and Territorial Factors.”

This revised title preserves the Reviewer’s proposed population description while replacing “determinants” with “associations with factors,” which is more consistent with the cross-sectional design.

**Reviewer 2 - Comment 3**

Line 125: Remove “either”.

**Response**

The word “either” was removed from the contextual description in the Methods. This grammatical revision improves sentence clarity without altering the substantive meaning.

**Reviewer 2 - Comment 4**

Lines 174-176: Information is repeated.

**Response**

The redundant passage was removed from the Methods. The outcome definition and its conceptual qualification are now presented once, in a structured sequence, in lines 162–179.

This revision reduces repetition while preserving the distinction between self-reported realized access, its contextual influences, and the secondary outcome of problem resolution.

**Reviewer 2 - Comment 5**

Use “Lasso” and “Ridge,” with only the first letter capitalized.

**Response**

The terminology was standardized as “Lasso” and “Ridge” throughout the Methods, Results, tables, and Discussion. This correction ensures consistent statistical nomenclature across the manuscript.

**Reviewer 2 - Comment 6**

Lines 202–203: Repeated information.

**Response**

The duplicated methodological statement was removed. The preprocessing, training, cross-validation, and test-set procedures are now described only once in the predictive-modeling subsection, lines 193-226.

This improves readability and avoids implying that different preprocessing procedures were used in separate stages.

**Reviewer 2 -Comment 7**

Replace “when class imbalance was present” with “In cases of class imbalance”.

**Response**

The sentence was revised in the model-evaluation subsection, lines 223–228, as recommended. The revised wording improves grammatical precision and preserves the methodological statement concerning class weighting and threshold evaluation.

**Reviewer 2 - Comment 8**

Replace the clustering sentence with: “Variables included in clustering were selected to reflect access-related characteristics; post-access outcomes were excluded, as the goal was to describe access profiles.”

**Response**

The recommended sentence was incorporated into the clustering subsection in the Methods, lines 229-235.

This wording clarifies that cluster construction was intended to describe access-related profiles rather than combine predictors with consequences occurring after access. It also strengthens the temporal coherence of the exploratory analysis.

**Reviewer 2 - Comment 9**

Replace with “was adapted for the healthcare access outcome”.

**Response**

The wording was revised as requested in the applicable methodological description. The change improves precision by indicating that the analytical procedure was specifically configured for the healthcare-access outcome.

**Reviewer 2 - Comment 10**

Replace with “lower socioeconomic status”.

**Response**

The expression was replaced by “lower socioeconomic status” in the PCA/SVI subsection, lines 242-249. This terminology is more precise and avoids potentially value-laden or ambiguous language.

**Reviewer 2 - Comment 11**

The statement about 517 records and 512 participants is repeated in the Methods and Results.

**Response**

The repeated sentence was removed from the Results. Information regarding the initial 517 records, exclusion of five observations with missing primary-outcome data, and final analytical sample of 512 participants is now reported only in the Methods, lines 180-192.

This preserves methodological transparency while removing duplication.

**Reviewer 2 - Comment 12**

Table 3 is unnecessary because the information is repeated in the text.

**Response**

We removed the previously redundant table and reorganized the numbering of the remaining tables. In the current manuscript, the model-comparison table is retained because it provides a comparative presentation of cross-validation and independent test-set metrics for all five models, while the text reports only the principal interpretation.

The Results text now emphasizes the limited discrimination and the sensitivity–specificity imbalance rather than reproducing every value. The complete comparative metrics remain available in the table for analytical transparency.

**Reviewer 2 - Comment 13**

Table 4 is unnecessary because all information is repeated in the text.

**Response**

We appreciate the concern regarding redundancy. We retained Table 4 because it provides the complete coefficients for the Lasso and Ridge models and permits direct comparison of the direction and relative magnitude of the associations across penalization methods.

The preceding Results paragraph, lines 326-332, was revised into an interpretative synthesis and no longer reproduces every coefficient. The numerical estimates are presented exclusively in Table 4, lines 333-339.

Thus, the table serves a distinct scientific function: it provides transparent model output, while the narrative explains its substantive interpretation.

**Reviewer 2 - Comment 14**

Change to “Lasso” and “Ridge”.

**Response**

The terms were standardized as “Lasso” and “Ridge” in the text and table title. This revision ensures consistent capitalization and statistical nomenclature.

**Reviewer 2 - Comment 15**

Table 7 is unnecessary because the information is repeated in the text.

**Response**

We retained Table 7 because the community random-intercept standard deviation, variance, and ICC are necessary for readers to evaluate the magnitude of contextual heterogeneity in the multilevel model. The duplicated numerical sentence was removed. The paragraph preceding the table, lines 361-367, now provides only the substantive interpretation that between-community residual variability was small after adjustment.

The exact variance parameters remain exclusively in Table 7, lines 368-372. This separation prevents repetition while preserving reproducibility and transparent multilevel reporting.

**Reviewer 2 - Comment 16**

Table 9 is unnecessary because all information is repeated in the text.

**Response**

We retained Table 9 because PCA interpretation requires disclosure of component loadings. Without the loadings, readers could not independently assess which variables defined PC1 or whether its interpretation as a vulnerability-related dimension was substantively justified. The narrative, lines 375-378, now states only that housing type and transportation contributed more strongly, while race/color had a near-zero loading. All exact numerical weights are presented exclusively in Table 9, lines 379-383.

This eliminates redundancy while maintaining methodological transparency.

**Reviewer 2 - Comment 17**

Line 393: “Lasso,” with only the first letter capitalized. Line 415: Correct to “context”.

**Response**

Both corrections were implemented. “Lasso” was standardized throughout the manuscript, and “context” was corrected in the Discussion. These changes improve linguistic and terminological consistency without affecting the scientific interpretation.

**Reviewer 2 - Comment 18**

Replace “assumes more dramatic contours” with “becomes more severe”.

**Response**

The expression was replaced with “becomes more severe” in the Discussion. The revised formulation is more objective and appropriate for scientific writing because it avoids figurative language.

**Reviewer 2 - Comment 19**

Why were telehealth services not discussed in greater depth? This topic is highly relevant to the Amazon region.

Response

We thank the Reviewer and agree that telehealth is highly relevant to healthcare access in geographically isolated Amazonian territories.

The Discussion was expanded to address telehealth as a potential strategy for: specialist consultation; clinical decision support; longitudinal follow-up; health education; integration with primary and fluvial healthcare teams.

We also discuss the structural conditions that may limit implementation, including unstable internet access, irregular electricity, limited digital inclusion, and insufficient technological infrastructure.

However, telehealth was not included as an exposure or service-use variable in the present analytical dataset, and the study was not designed to evaluate the availability, utilization, effectiveness, or acceptability of telehealth interventions. We therefore discuss telehealth as a policy and service-delivery implication rather than as an empirically tested explanation for the observed access patterns.

This approach gives the topic appropriate contextual prominence without implying that the present data directly evaluated telehealth services.

Beyond the specific comments, we made several additional changes to strengthen scientific precision. First, the study design was revised from “population-based” to “community-based cross-sectional survey”, because recruitment was non-probabilistic and depended on residents’ presence and availability during fieldwork. Second, the Abstract now describes the predictive performance as limited, noting a held-out test-set AUC range of 0.482–0.586 and poor generalizability for individual-level prediction. Third, the limitations now state that variable-importance measures describe the fitted models and should not be interpreted as causal effects. Fourth, the Conclusion was fully rewritten. It now states that: access varied across social, organizational, and territorial characteristics; the findings represent associations rather than causal effects; predictive discrimination was limited; machine learning contributed primarily to exploratory pattern recognition; community-level findings are descriptive; future longitudinal, multisite, and probabilistically sampled studies are required.

We sincerely thank the Editor and Reviewers for their thoughtful and rigorous assessment. Their comments led us to improve the conceptual definition of the outcome, align the study objective with the cross-sectional design, clarify the sampling strategy, moderate the interpretation of predictive and contextual analyses, expand the limitations, and improve consistency across all submission documents.

We believe that the revised manuscript now presents a more transparent, methodologically balanced, and scientifically defensible account of healthcare-access inequalities in the quilombola rural and riverside communities investigated.

Sincerely,

Lívia de Aguiar Valentim
Corresponding author
On behalf of all authors
