# Supplementary material for: Healthcare access inequalities in Quilombola rural and riverside communities in the Brazilian Amazon: Associations with social, organizational, and territorial factors
Source: PLoS One. 2026 Aug 10;21(8):e0355489. doi: 10.1371/journal.pone.0355489 (PMC13456258; doi:10.1371/journal.pone.0355489)
Supplement: Peer review history

# Peer review: Decision Letter 0

**DOI:** 10.1371/journal.pone.0355489.r001

**Authors:** Angelica Miranda

6 May 2026

Dear Dr.  Valentim,

Thank you for submitting your manuscript to PLOS ONE. After careful consideration, we feel that it has merit but does not fully meet PLOS ONE’s publication criteria as it currently stands. Therefore, we invite you to submit a revised version of the manuscript that addresses the points raised during the review process.

Please submit your revised manuscript by Jun 20 2026 11:59PM. If you will need more time than this to complete your revisions, please reply to this message or contact the journal office at plosone@plos.org. When you're ready to submit your revision, log on to https://www.editorialmanager.com/pone/ and select the 'Submissions Needing Revision' folder to locate your manuscript file.

- A letter that responds to each point raised by the academic editor and reviewer(s). You should upload this letter as a separate file labeled 'Response to Reviewers'.
- A marked-up copy of your manuscript that highlights changes made to the original version. You should upload this as a separate file labeled 'Revised Manuscript with Track Changes'.
- An unmarked version of your revised paper without tracked changes. You should upload this as a separate file labeled 'Manuscript'.

If you would like to make changes to your financial disclosure, please include your updated statement in your cover letter. Guidelines for resubmitting your figure files are available below the reviewer comments at the end of this letter.

If applicable, we recommend that you deposit your laboratory protocols in protocols.io to enhance the reproducibility of your results. Protocols.io assigns your protocol its own identifier (DOI) so that it can be cited independently in the future. For instructions see: https://journals.plos.org/plosone/s/submission-guidelines#loc-laboratory-protocols. Additionally, PLOS ONE offers an option for publishing peer-reviewed Lab Protocol articles, which describe protocols hosted on protocols.io. Read more information on sharing protocols at https://plos.org/protocols?utm_medium=editorial-email&utm_source=authorletters&utm_campaign=protocols.

As the corresponding author, your ORCID iD is verified in the submission system and will appear in the published article. PLOS supports the use of ORCID, and we encourage all coauthors to register for an ORCID iD and use it as well. Please encourage your coauthors to verify their ORCID iD within the submission system before final acceptance, as unverified ORCID iDs will not appear in the published article. *Only*  the individual author can complete the verification step; PLOS staff *cannot*  verify ORCID iDs on behalf of authors.

We look forward to receiving your revised manuscript.

Kind regards,

Angelica Espinosa Miranda, M.D., Ph.D.

Academic Editor

PLOS One

Journal Requirements:

When submitting your revision, we need you to address these additional requirements.

1.Please ensure that your manuscript meets PLOS ONE's style requirements, including those for file naming. The PLOS ONE style templates can be found at

https://journals.plos.org/plosone/s/file?id=wjVg/PLOSOne_formatting_sample_main_body.pdf and

https://journals.plos.org/plosone/s/file?id=ba62/PLOSOne_formatting_sample_title_authors_affiliations.pdf

2. Thank you for your submission to PLOS One. We note that your cover letter mentions an article with the title "“Territorial Determinants, Social Vulnerability, and Healthcare Access in Quilombola Communities in the Brazilian Amazon", but the main manuscript's title is "Healthcare Access Inequalities in Quilombola, Rural, and Riverside Communities in the Brazilian Amazon: Social, Organizational, and Territorial Determinants". Can you please clarify which is the correct title and check if you have submitted an incorrect cover letter or incorrect manuscript file? If one is incorrect please upload the correct file.

3. Please note that PLOS One has specific guidelines on code sharing for submissions in which author-generated code underpins the findings in the manuscript. In these cases, we expect all author-generated code to be made available without restrictions upon publication of the work. Please review our guidelines at https://journals.plos.org/plosone/s/materials-and-software-sharing#loc-sharing-code and ensure that your code is shared in a way that follows best practice and facilitates reproducibility and reuse.

4. We note that you have indicated that there are restrictions to data sharing for this study. PLOS only allows data to be available upon request if there are legal or ethical restrictions on sharing data publicly. For more information on unacceptable data access restrictions, please see http://journals.plos.org/plosone/s/data-availability#loc-unacceptable-data-access-restrictions.

Before we proceed with your manuscript, please address the following prompts:

a) If there are ethical or legal restrictions on sharing a de-identified data set, please explain them in detail (e.g., data contain potentially identifying or sensitive patient information, data are owned by a third-party organization, etc.) and who has imposed them (e.g., a Research Ethics Committee or Institutional Review Board, etc.). Please also provide contact information for a data access committee, ethics committee, or other institutional body to which data requests may be sent.

b) If there are no restrictions, please upload the minimal anonymized data set necessary to replicate your study findings to a stable, public repository and provide us with the relevant URLs, DOIs, or accession numbers. For a list of recommended repositories, please see

https://journals.plos.org/plosone/s/recommended-repositories. You also have the option of uploading the data as Supporting Information files, but we would recommend depositing data directly to a data repository if possible.

We will update your Data Availability statement on your behalf to reflect the information you provide.

5. Thank you for stating the following in the Competing Interests section:

“NO authors have competing interests”

We note that one or more of the authors are employed by a commercial company: Academic of the Metropolitan College of Pará – FAMETRO, Santarém, Pará, Brasil and Academic of the University of Amazonia – UNAMA, Santarém, Pará, Brasil

1.        Please provide an amended Funding Statement declaring this commercial affiliation, as well as a statement regarding the Role of Funders in your study. If the funding organization did not play a role in the study design, data collection and analysis, decision to publish, or preparation of the manuscript and only provided financial support in the form of authors' salaries and/or research materials, please review your statements relating to the author contributions, and ensure you have specifically and accurately indicated the role(s) that these authors had in your study. You can update author roles in the Author Contributions section of the online submission form.

Please also include the following statement within your amended Funding Statement.

“The funder provided support in the form of salaries for authors [insert relevant initials], but did not have any additional role in the study design, data collection and analysis, decision to publish, or preparation of the manuscript. The specific roles of these authors are articulated in the ‘author contributions’ section.”

If your commercial affiliation did play a role in your study, please state and explain this role within your updated Funding Statement.

2. Please also provide an updated Competing Interests Statement declaring this commercial affiliation along with any other relevant declarations relating to employment, consultancy, patents, products in development, or marketed products, etc.

Within your Competing Interests Statement, please confirm that this commercial affiliation does not alter your adherence to all PLOS ONE policies on sharing data and materials by including the following statement: "This does not alter our adherence to  PLOS ONE policies on sharing data and materials.” (as detailed online in our guide for authors http://journals.plos.org/plosone/s/competing-interests) . If this adherence statement is not accurate and  there are restrictions on sharing of data and/or materials, please state these. Please note that we cannot proceed with consideration of your article until this information has been declared.

Please include both an updated Funding Statement and Competing Interests Statement in your cover letter. We will change the online submission form on your behalf.

6. Your ethics statement should only appear in the Methods section of your manuscript. If your ethics statement is written in any section besides the Methods, please move it to the Methods section and delete it from any other section. Please ensure that your ethics statement is included in your manuscript, as the ethics statement entered into the online submission form will not be published alongside your manuscript.

7. If the reviewer comments include a recommendation to cite specific previously published works, please review and evaluate these publications to determine whether they are relevant and should be cited. There is no requirement to cite these works unless the editor has indicated otherwise.

Reviewers' comments:

Reviewer's Responses to Questions

**Comments to the Author**

1. Is the manuscript technically sound, and do the data support the conclusions?

Reviewer #1: Yes

Reviewer #2: Yes

**********

2. Has the statistical analysis been performed appropriately and rigorously?

Reviewer #1: Yes

Reviewer #2: Yes

**********

3. Have the authors made all data underlying the findings in their manuscript fully available?

The PLOS Data policy

Reviewer #1: Yes

Reviewer #2: Yes

**********

4. Is the manuscript presented in an intelligible fashion and written in standard English?

Reviewer #1: Yes

Reviewer #2: Yes

**********

Reviewer #1: This manuscript addresses a relevant and timely topic, focusing on healthcare access inequalities in vulnerable populations in the Brazilian Amazon, particularly among quilombola, rural, and riverside communities. The use of primary data collected in hard-to-reach settings represents an important strength, and the attempt to integrate individual, organizational, and territorial dimensions is commendable. However, despite its relevance, the manuscript presents important conceptual, methodological, and reporting limitations that affect the interpretability and robustness of the findings. Recommendation: Major Revision Substantial revisions are required to improve conceptual clarity, methodological justification, and adherence to reporting standards such as the: STROBE Statement. Major Comments: 1 - Misalignment between the study objective and design. The manuscript aims to “identify determinants” of healthcare access. However, given the cross-sectional design, this wording overstates the analytical capacity of the study. Cross-sectional studies do not establish temporality and are not suited to identifying determinants in a causal sense. The objective should be revised to reflect the exploratory and associative nature of the analysis. 2- Conceptual fragility of the primary outcome (“healthcare access”)

The outcome is based on self-reported receipt of care when needed, which may conflate need, demand, and access, and is subject to recall and reporting bias. A stronger conceptual framework is required to support this operationalization and avoid overinterpretation.3- Insufficient discussion of potential biases Key sources of bias are not adequately addressed, including: selection bias (community-based recruitment), information bias (self-reported measures).Minor Comments (methodologically relevant): 4- Absence of descriptive table of sample characteristics: The Results section lacks a comprehensive table describing the study population. This limits interpretability and assessment of external validity. Inclusion is recommended in accordance with the STROBE Statement. 5 - Potential overinterpretation of community-level findings: Findings based on aggregated data should be interpreted cautiously, considering the limited number of communities and the risk of ecological inference.

Final Assessment: The manuscript addresses an important public health issue and is based on valuable primary data. However, the main limitation lies in the misalignment between the complexity of the analytical approach and the conceptual and methodological consistency of the study. At present, the study appears to overextend its conclusions relative to its design and analytical capacity. Addressing these issues—particularly regarding the study objective, outcome definition, analytical coherence, and reporting transparency—is essential for improving the scientific contribution of the manuscript.

Reviewer #2: This seems to me a very important study, as it reveals a reality of the difficulties that exist in the Amazon region.

It is necessary to include a map that contextualizes the locations of the communities used in the study, to give more meaning to their location and to better understand how isolated these communities are, not everyone is familiar with the reality of the Amazon region.

I would recommend changing the title to: "Healthcare Access Inequalities in Rural and Riverside Quilombola Communities in the Brazilian Amazon: Social, Organizational, and Territorial Determinants". With the original title, it seems as if they were going to analyze three types of communities (quilombola, rural, and riverside) separately, but upon reading the methodology, it becomes clear that they refer to the characteristics of a single community.

Line 125: Remove "either"

Lines 174-176: Repeated information from lines 161-164.

Line 193: Lasso and Ridge (only the first letter capitalized).

Lines 202-203: Repetition of information from lines 200-201.

Line 205: Replace "when class imbalance was present" with "In cases of class imbalance"

Lines 211-212: Replace with "Variables included in clustering were selected to reflect access-related characteristics; post-access outcomes were excluded, as the goal was to describe access profiles"

Line 215: Replace with "was fitted for the outcome of healthcare access"

Line 223: Replace with "lower socioeconomic status"

Line 234: "Among the 517 records in the database, 512 participants". this information already appears in the methodology, in lines 161-164 and 174-176.

Line 277: Table 3 is unnecessary because all that information is already repeated in the text, described in lines 267-275.

Line 288: Table 4 is unnecessary because all that information is already repeated in the text, described in lines 279-286. Either include only the most important information in the text, or remove the table.

Line 281: Change to "Lasso" and "Ridge"

Line 322: Table 7 is unnecessary because all that information is already repeated in the text, described in lines 315-321.

Line 336: Table 9 is unnecessary because all that information is already repeated in the text, described in lines 330-334. Either include only the most important information in the text, or remove the table.

Line 393: Lasso, only the first letter capitalized.

Line 415: Correct to "context"

Line 429: Replace "assumes more dramatic contours" with "becomes more severe"

Question: I would like to know why telehealth services were not discussed further. There is much articles on this topic, even within the Amazon. It is only mentioned in line 475. In my opinion, this is something that should be discussed given the subject matter.

**********

what does this mean?). If published, this will include your full peer review and any attached files.

If you choose “no”, your identity will remain anonymous but your review may still be made public.

**Do you want your identity to be public for this peer review?**  For information about this choice, including consent withdrawal, please see our Privacy Policy

Reviewer #1: No

Reviewer #2: **Yes:**  Susan Smith-Doria

**********

[NOTE: If reviewer comments were submitted as an attachment file, they will be attached to this email and accessible via the submission site. Please log into your account, locate the manuscript record, and check for the action link "View Attachments". If this link does not appear, there are no attachment files.]

To ensure your figures meet our technical requirements, please review our figure guidelines: https://journals.plos.org/plosone/s/figures

You may also use PLOS’s free figure tool, NAAS, to help you prepare publication quality figures: https://journals.plos.org/plosone/s/figures#loc-tools-for-figure-preparation.

NAAS will assess whether your figures meet our technical requirements by comparing each figure against our figure specifications.

# Author comment: Author response to Decision Letter 1

**DOI:** 10.1371/journal.pone.0355489.r002

16 Jun 2026

Data Availability Statement

R: There are no ethical or legal restrictions preventing the sharing of the anonymized dataset used in this study. The dataset has been fully anonymized prior to public release and does not contain any direct identifiers or information that could reasonably lead to participant identification. The study was approved by the Research Ethics Committee of the State University of Pará (UEPA), approval number 4.915.684, and all procedures complied with Brazilian ethical regulations and the Declaration of Helsinki.

The minimum dataset required to reproduce the analyses reported in this manuscript, together with the corresponding codebook describing all variables and coding procedures, is publicly available and may be accessed through the following DOI: https://doi.org/10.1371/journal.pntd.0014078.s001

This repository contains the anonymized dataset supporting the findings of the study, including sociodemographic, environmental, territorial, and health-related variables used in the statistical and machine-learning analyses. Therefore, there are no restrictions on data access, and no data access committee is required. All interested researchers may freely access the dataset through the DOI provided above.

Funding Statement

R: This study received no specific funding from any public, commercial, or not-for-profit funding agency. Although some co-authors are undergraduate medical students affiliated with Metropolitan College of Pará – FAMETRO, Santarém, Pará, Brasil and University of Amazonia – UNAMA, Santarém, Pará, Brasil, these institutions did not provide financial support, salaries, research materials, infrastructure, or any other resources for this study. The research was conducted within the framework of the State University of Pará (UEPA), a public university. The participation of students from FAMETRO and UNAMA occurred on a voluntary basis and was unrelated to any institutional sponsorship, employment relationship, or financial support. Therefore, no commercial entity had any role in the study design, data collection, data analysis, decision to publish, or preparation of the manuscript.

Competing Interests Statement

The authors declare that they have no competing interests. The editorial office identified affiliations with Metropolitan College of Pará – FAMETRO, Santarém, Pará, Brasil and University of Amazonia – UNAMA, Santarém, Pará, Brasil as potential commercial affiliations. However, the authors affiliated with these institutions are undergraduate students and are not employees, consultants, shareholders, or representatives of these organizations. These institutions did not provide funding, salaries, materials, infrastructure, or any form of support for this research. The participation of these authors was entirely voluntary and independent of their academic affiliations. This does not alter our adherence to PLOS ONE policies on sharing data and materials.

REVIEWER 1

1. Misalignment between the study objective and study design. The manuscript aims to “identify determinants” of healthcare access. However, given the cross-sectional design, this wording overstates the analytical capacity of the study. Cross-sectional studies do not establish temporality and are not suitable for identifying determinants in a causal sense. The objective should be revised to reflect the exploratory and associative nature of the analysis.

Response: We thank the reviewer for this observation. We clarify that we consider healthcare access as a multidimensional and territorially conditioned phenomenon, a concept derived from the Social Determinants of Health framework widely used in health research, particularly in the Amazon region and strongly linked to the healthcare system adopted in Brazil. To improve methodological precision and avoid causal interpretations, we revised the study objective by replacing the verb “identify” with “investigate.” The objective now reads: “The present study aimed to investigate social and territorial determinants of healthcare access in the Brazilian Amazon.”

2. Conceptual weakness of the primary outcome (“healthcare access”). The outcome is based on self-reported receipt of care when needed, which may conflate need, demand, and access and is subject to recall and reporting biases. A more robust conceptual framework is needed to support this operationalization and avoid overinterpretation.

Response: To provide a more robust conceptual framework regarding the theoretical conception of access adopted in this study, we revised the manuscript and added the following text to the Methods section (lines 151–159): “This approach was adopted considering the organization of health services in the Amazon region and the geographical and organizational characteristics of the communities studied, which are related to the effective provision of care in the context of the healthcare system. In this sense, access no longer depends exclusively on spontaneous demand or individual perception of need, but rather on the organization and interrelation of services, referral and counter-referral processes, the implementation of active search strategies, the presence of a multidisciplinary team in the territory, and the ability to travel to access or continue using the healthcare network.”

3. Insufficient discussion of potential biases. Major sources of bias are not adequately addressed, including selection bias (community-based recruitment) and information bias (self-reported measures).

Response: We thank the reviewer for this observation. We expanded the discussion of methodological biases present in the study, with particular emphasis on information bias. In addition, we detailed the possible implications of recall bias, social desirability bias, and community-based recruitment. The following sections were revised: “Second, several variables were based on self-reported information, which may be subject to recall bias, leading to the oversimplification or underreporting of older events, an overemphasis on more memorable experiences at the expense of routine healthcare visits, and distorted perceptions resulting from the fragmented care context experienced in the region. In addition, they may be influenced by social desirability bias, stemming from embarrassment when reporting information, possible influence by interviewers, and the historical context of vulnerability of the studied population, generating fear of negative consequences for the community or personal relationships with the health service.”

“The study was conducted in quilombola communities within a specific region of the Brazilian Amazon, whose territorial and logistical characteristics (population dispersion, seasonal and precarious displacement, high-cost transportation, and geographic isolation) increase the complexity of fieldwork and hinder population recruitment...”

4. Absence of a descriptive table of sample characteristics. The Results section does not present a comprehensive table describing the study population. This limits interpretability and the assessment of external validity. Inclusion is recommended according to the STROBE Statement.

Response: A table describing the sociodemographic characteristics of the analyzed sample was included.

5. Potential overinterpretation of community-level results. Results based on aggregated data should be interpreted cautiously, considering the limited number of communities and the risk of ecological inference.

Response: We thank the reviewer for this observation. We revised the Discussion and Conclusion sections to provide a more cautious interpretation of the findings at the community level, correcting passages that could suggest extrapolation or generalization beyond the inferential scope of the study. We also reinforced the study limitations to make clear the need for caution when interpreting and generalizing the findings.

REVIEWER 2

1. It is necessary to include a map contextualizing the location of the communities used in the study, to provide a better understanding of how isolated these communities are, since not all readers are familiar with the Amazonian reality.

Response: A map was included to contextualize the analyzed communities.

2. I would recommend changing the title to: “Healthcare Access Inequalities in Quilombola Rural and Riverside Communities in the Brazilian Amazon: Social, Organizational, and Territorial Determinants.” With the original title, it appears that three different community types (quilombola, rural, and riverside) are being analyzed separately, whereas the methodology makes it clear that these characteristics refer to the same communities.

Response: We thank the reviewer for the suggestion. The title was revised and changed to: “Healthcare Access Inequalities in Quilombola Rural and Riverside Communities in the Brazilian Amazon: Social, Organizational, and Territorial Determinants.”

3. Line 125: Remove “either”.

Response: Removed.

4. Lines 174–176: Information repeated from lines 161–164.

Response: Verified and redundant information was removed.

5. Line 193: Lasso and Ridge (only the first letter capitalized).

Response: Verified and corrected.

6. Lines 202–203: Repetition of information from lines 200–201.

Response: Verified and redundant information was removed.

7. Line 205: Replace “when class imbalance was present” with “In cases of class imbalance”.

Response: Verified and replaced.

8. Lines 211–212: Replace with “Variables included in clustering were selected to reflect access-related characteristics; post-access outcomes were excluded, as the goal was to describe access profiles.”

Response: Verified and replaced.

9. Line 215: Replace with “was adapted for the healthcare access outcome”.

Response: Verified and replaced.

10. Line 223: Replace with “lower socioeconomic status”.

Response: Verified and replaced.

11. Line 234: “Among the 517 records in the database, 512 participants...” This information already appears in the Methods section.

Response: Verified and lines 234–235 were removed.

12. Line 277: Table 3 is unnecessary because the information is repeated in the text.

Response: Verified and Table 3 was removed.

13. Line 288: Table 4 is unnecessary because all information is repeated in the text.

Response: We chose to retain the table and revise the preceding text into an interpretative synthesis of the findings, avoiding repetition of coefficients already presented in the table and reducing redundancy.

14. Line 281: Change to “Lasso” and “Ridge”.

Response: Verified and corrected.

15. Line 322: Table 7 is unnecessary because all information is repeated in the text.

Response: We chose to retain the table and removed the sentence “The community-level random-intercept standard deviation was 0.201 (variance = 0.040), corresponding to an approximate ICC of 0.012,” maintaining the interpretation of the findings while avoiding duplication.

16. Line 336: Table 9 is unnecessary because all information is repeated in the text.

Response: Numerical values and the phrase “with additional contributions from education (−0.424), employment status (−0.349), and household income (−0.335)” were removed from the text preceding Table 9, maintaining the interpretation while avoiding repetition.

17. Line 393: Lasso, only the first letter capitalized.

Line 415: Correct to “context”.

Response: Verified and corrected.

18. Line 429: Replace “assumes more dramatic contours” with “becomes more severe”.

Response: Verified and replaced.

19. Question: I would like to know why telehealth services were not discussed in greater depth. There are many articles on this topic, including in the Amazon region itself. It is mentioned only briefly. In my opinion, this topic should be addressed given its relevance.

Response: We thank the reviewer for this observation and recognize the relevance of telehealth as a strategy to expand healthcare access, particularly in the Brazilian Amazon. We added a paragraph addressing this issue. However, we chose not to discuss it in greater depth because telehealth/telemedicine services were not available in the communities investigated during the study period and were not identified by participants as part of their healthcare access experience. Therefore, although the topic is relevant to the broader discussion, it was not part of the investigation or analytical framework of the present study and was mentioned only as a potential facilitator of healthcare access.

Attachment Submitted filename: LETTER OF REPLY.pdf

# Editor report: Decision Letter 1

**DOI:** 10.1371/journal.pone.0355489.r003

**Authors:** Angelica Miranda

9 Jul 2026

Dear Dr. Valentim,

Thank you for submitting your manuscript to PLOS ONE. After careful consideration, we feel that it has merit but does not fully meet PLOS ONE’s publication criteria as it currently stands. Therefore, we invite you to submit a revised version of the manuscript that addresses the points raised during the review process.

Please submit your revised manuscript by Aug 23 2026 11:59PM. If you will need more time than this to complete your revisions, please reply to this message or contact the journal office at plosone@plos.org. When you're ready to submit your revision, log on to https://www.editorialmanager.com/pone/ and select the 'Submissions Needing Revision' folder to locate your manuscript file.

- A letter that responds to each point raised by the academic editor and reviewer(s). You should upload this letter as a separate file labeled 'Response to Reviewers'.
- A marked-up copy of your manuscript that highlights changes made to the original version. You should upload this as a separate file labeled 'Revised Manuscript with Track Changes'.
- An unmarked version of your revised paper without tracked changes. You should upload this as a separate file labeled 'Manuscript'.

If you would like to make changes to your financial disclosure, please include your updated statement in your cover letter. Guidelines for resubmitting your figure files are available below the reviewer comments at the end of this letter.

If applicable, we recommend that you deposit your laboratory protocols in protocols.io to enhance the reproducibility of your results. Protocols.io assigns your protocol its own identifier (DOI) so that it can be cited independently in the future. For instructions see: https://journals.plos.org/plosone/s/submission-guidelines#loc-laboratory-protocols. Additionally, PLOS ONE offers an option for publishing peer-reviewed Lab Protocol articles, which describe protocols hosted on protocols.io. Read more information on sharing protocols at https://plos.org/protocols?utm_medium=editorial-email&utm_source=authorletters&utm_campaign=protocols.

As the corresponding author, your ORCID iD is verified in the submission system and will appear in the published article. PLOS supports the use of ORCID, and we encourage all coauthors to register for an ORCID iD and use it as well. Please encourage your coauthors to verify their ORCID iD within the submission system before final acceptance, as unverified ORCID iDs will not appear in the published article. *Only*  the individual author can complete the verification step; PLOS staff *cannot*  verify ORCID iDs on behalf of authors.

We look forward to receiving your revised manuscript.

Kind regards,

Angelica Espinosa Miranda, M.D., Ph.D.

Academic Editor

PLOS One

Journal Requirements:

If the reviewer comments include a recommendation to cite specific previously published works, please review and evaluate these publications to determine whether they are relevant and should be cited. There is no requirement to cite these works unless the editor has indicated otherwise.

**Additional Editor Comments:**

Thank you for submitting the revised version of your manuscript entitled "Healthcare Access Inequalities in Quilombola, Rural, and Riverside Communities in the Brazilian Amazon: Social, Organizational, and Territorial Determinants."

Both reviewers acknowledge the relevance of the study and recognize that substantial improvements have been made in response to the initial round of peer review. In particular, the manuscript now provides a clearer description of the analytical methods, includes additional contextual information, and addresses several concerns regarding reporting and presentation. However, after evaluating the revised manuscript and the accompanying response letter, I believe that several reviewer comments have only been partially addressed. Before the manuscript can be considered further, I ask the authors to carefully review the remaining points below.

1. Ensure that every reviewer comment is fully addressed. For several comments, the response letter states that a change was made (e.g., "verified and corrected", "revised", or "removed"), but the revision does not always fully resolve the underlying concern raised by the reviewer. Please ensure that each response addresses not only the requested textual modification but also the scientific rationale behind the reviewer's concern. For example: the conceptual definition of healthcare access should be strengthened by explicitly acknowledging the limitations of the operational definition used in the study; limitations inherent to the cross-sectional design should be discussed more explicitly, particularly regarding the inability to establish temporality or causal relationships.

2. Responses should explain why, not only what. Several responses simply indicate that a modification was performed without explaining how the concern was addressed. Please revise the response letter so that each response clearly explains:

what changes were made; where these changes can be found in the manuscript; how these changes address the reviewer's concern. This will greatly facilitate editorial assessment.

3. When a reviewer suggestion is not adopted, provide a scientific justification. The reviewers made some recommendations that the authors elected not to follow (for example, regarding the removal of certain tables). Authors are not required to implement every suggestion. However, whenever a recommendation is not adopted, the response should provide a clear scientific justification rather than simply stating that the table or text was retained.

4. Verify consistency throughout the manuscript. Please carefully verify that all terminology modified in response to reviewer comments has been updated consistently throughout the manuscript. In particular, ensure that revisions made to the study objective are reflected consistently in: the Abstract; the Introduction; the manuscript metadata; the cover letter; and the response letter. Consistency across all submitted documents is important.

5. Data Availability Statement. Please carefully review the revised Data Availability Statement.

If the dataset is now publicly available, ensure that: the repository corresponds to the dataset used in the present study; the statement in the manuscript is fully consistent with the information provided in the submission system. If data availability has changed since the original submission, please ensure that this change is clearly explained. In the initial submission the authors stated: "data cannot be shared publicly...". In the reviewers response the answer changed to "there are no ethical restrictions...". It will be important explain it further.

6. Final review of limitations. Although the limitations section has been expanded, I encourage the authors to ensure that it explicitly discusses: possible information bias associated with self-reported variables; potential selection bias related to community-based recruitment; the limited number of communities included in contextual analyses and the implications for generalizability. A balanced discussion of these issues will strengthen the manuscript.

Overall, the text improved positively, and I believe the manuscript has the potential to make a valuable contribution to the literature. Addressing the remaining issues above should substantially improve its scientific clarity and facilitate the final editorial decision. I look forward to receiving a carefully revised manuscript together with a detailed point-by-point response explaining how each remaining issue has been addressed.

[NOTE: If reviewer comments were submitted as an attachment file, they will be attached to this email and accessible via the submission site. Please log into your account, locate the manuscript record, and check for the action link "View Attachments". If this link does not appear, there are no attachment files.]

To ensure your figures meet our technical requirements, please review our figure guidelines: https://journals.plos.org/plosone/s/figures

You may also use PLOS’s free figure tool, NAAS, to help you prepare publication quality figures: https://journals.plos.org/plosone/s/figures#loc-tools-for-figure-preparation.

NAAS will assess whether your figures meet our technical requirements by comparing each figure against our figure specifications.

# Author comment: Author response to Decision Letter 2

**DOI:** 10.1371/journal.pone.0355489.r004

13 Jul 2026

RESPONSE TO THE EDITOR AND REVIEWERS

Manuscript title: Healthcare Access Inequalities in Quilombola Rural and Riverside Communities in the Brazilian Amazon: Associations with Social, Organizational, and Territorial Factors

Dear Editor and Reviewers,

We sincerely thank the Editor and the Reviewers for the careful re-evaluation of our manuscript and for the detailed and constructive recommendations. We appreciate the recognition that the previous revision improved the methodological description, contextualization, and presentation of the findings.

We agree with the Editor that some of our previous responses described the changes made without sufficiently explaining the scientific rationale underlying those changes. We therefore conducted a comprehensive revision of both the manuscript and the response letter. In the present response, each item specifies: (1) what was changed; (2) where the change can be found; and (3) how the modification addresses the methodological, conceptual, or editorial concern raised.

All changes have been highlighted in the revised manuscript.

ADDITIONAL COMMENTS FROM THE EDITOR

Editor comment 1:

Please ensure that all reviewer comments are addressed in full. In several comments, the response letter states that a change was made, but the revision does not always fully resolve the underlying concern. In particular, the conceptual definition of healthcare access should be strengthened while explicitly acknowledging the limitations of the operational definition, and the limitations of the cross-sectional design should be discussed more explicitly regarding temporality and causality.

Response:

We thank the Editor for this important observation. We agree that our previous response did not fully distinguish the broad theoretical construct of healthcare access from the narrower operational measure used in this study.

Three substantive changes were made.

First, we reformulated the objective so that it no longer suggests causal identification. The revised objective now reads:

“The present study aimed to examine individual, organizational, and territorial factors associated with self-reported healthcare access among quilombola rural and riverside communities in Santarém, Brazilian Amazon, using conventional statistical and machine-learning approaches.”

This wording appears in the Abstract, lines 22–26, and at the end of the Introduction, lines 108–113. The terms “examine” and “associated with” were selected because the cross-sectional design permits the examination of associations and exploratory patterns but does not establish temporal ordering or causal determination.

Second, we substantially revised the operational definition of the primary outcome in the Methods, lines 162–179. Healthcare access is now explicitly defined as self-reported realized access, namely, receipt of healthcare after the participant perceived a need during the previous 12 months. We also state that this measure captures only one stage of the access process and does not represent a comprehensive assessment of healthcare accessibility. The manuscript now explicitly acknowledges that the measure does not fully distinguish health need, perceived need, demand, care-seeking, service availability, utilization, timeliness, continuity, or adequacy of care.

Third, we expanded the limitations in the Discussion, lines 485–527, to explain that the cross-sectional design precludes establishing whether the social, territorial, and organizational characteristics preceded or resulted from the reported access experience. We therefore state that the findings must be interpreted as associations and hypothesis-generating patterns rather than causal effects.

These revisions address the Editor’s concern by aligning the conceptual interpretation of the outcome and the reported findings with the actual measurement strategy and the inferential limits of the study design.

Editor comment 2

The responses should explain why, and not only what. Each response should clearly explain what changes were made, where the changes can be found, and how they address the reviewer’s concern.

Response

We agree and have completely restructured the response letter. Each response now includes: the specific textual, methodological, or analytical modification; the section and current line numbers in which the modification appears; an explanation of why the modification resolves or mitigates the concern raised.

Responses that previously stated only “verified,” “corrected,” “removed,” or “revised” have been replaced with complete explanations. For minor grammatical corrections, we identify the revised passage and explain that the modification improves linguistic accuracy or removes redundancy without altering the substantive meaning. For methodological issues, we explicitly describe the inferential or conceptual implications of the revision.

Editor comment 3

When a reviewer’s recommendation is not followed, provide a scientific justification. The authors are not required to implement every suggestion, but retaining a table or text should be scientifically justified.

Response

We thank the Editor for this clarification. We have expanded the responses concerning Tables 4, 7, and 9, which were retained despite the Reviewer’s recommendations for removal.

Table 4 was retained because it reports the complete coefficients from the Lasso and Ridge models and enables readers to evaluate the direction, relative magnitude, and consistency of the associations across the two penalized approaches. The accompanying Results text was shortened so that it now provides only an interpretative synthesis rather than repeating all coefficients. The revised text and table are located in the Results, lines 326-339.

Table 7 was retained because reporting the community-level random-intercept standard deviation, variance, and approximate ICC is necessary for transparent evaluation of contextual heterogeneity. These parameters cannot be adequately evaluated from a narrative statement alone. Numerical duplication was removed from the preceding paragraph, which now focuses on substantive interpretation. The revised paragraph and table are located in the Results, lines 361-372.

Table 9 was retained because interpretation and reproducibility of a PCA-derived index require transparent reporting of component loadings. The loadings show how each socioeconomic and structural variable contributed to the first component and allow readers to assess the substantive meaning of the Social Vulnerability Index. Numerical values were removed from the narrative and retained only in the table. The revised material appears in the Results, lines 375-383.

These decisions preserve the information necessary for analytical transparency while eliminating textual duplication.

Editor comment 4

Please verify consistency throughout the manuscript. In particular, revisions to the study objective should be reflected consistently in the Abstract, Introduction, manuscript metadata, cover letter, and response letter.

Response

We performed a systematic terminology review across the manuscript and accompanying submission documents.

The study objective has been standardized as:

“To examine individual, organizational, and territorial factors associated with self-reported healthcare access among quilombola rural and riverside communities in Santarém, Brazilian Amazon, using conventional statistical and machine-learning approaches.”

This wording appears in the Abstract, lines 22-26, and the Introduction, lines 108–113, and has also been incorporated into the revised cover letter, submission-system metadata, and response letter.

We also revised the study-design terminology. The study is now described in the Methods, lines 114–120, as a: “community-based cross-sectional survey”, rather than a population-based study. The same wording has been introduced in the Abstract.

Throughout the Results, Discussion, and Conclusion, causal formulations were revised, particularly when referring to the empirical findings. Terms such as “caused,” “determined,” “proved,” and “confirmed” were replaced, where appropriate, by “was associated with,” “showed a pattern,” “suggested,” or “was consistent with.”

The Conclusion, lines 528-536, now explicitly states that the findings represent associations and exploratory patterns and do not establish temporal or causal relationships.

The revised title was also aligned with this interpretation: “Healthcare Access Inequalities in Quilombola Rural and Riverside Communities in the Brazilian Amazon: Associations with Social, Organizational, and Territorial Factors.”

This terminology audit ensures consistency between the study objective, design, outcome definition, analytical strategy, and inferential claims.

Editor comment 5

Please carefully review the revised Data Availability Statement. If the dataset is now publicly available, ensure that the repository corresponds to the dataset used in the present study and that the statement is consistent with the submission system. If availability changed since the original submission, clearly explain the change.

Response

We thank the Editor for identifying the need to clarify the change in the Data Availability Statement. In the original submission, we adopted a conservative position regarding public data sharing because the study involved participants from small and geographically identifiable quilombola communities. We were particularly concerned about the possibility of indirect identification through combinations of territorial, demographic, and socioeconomic variables, even after the removal of direct personal identifiers.

Following the first review round, the dataset was reassessed and a minimum analytical dataset was prepared for public sharing. Direct identifiers were removed, and variables presenting a potential risk of indirect identification were reviewed, recoded, or organized in a manner intended to preserve participant confidentiality. The resulting dataset contains the observations and analytical variables required to support the findings reported in the manuscript, together with a codebook describing variable definitions, categories, and coding procedures.

The Data Availability Statement was revised in the manuscript, lines 575-584, and the same wording has been entered into the journal submission system. The revised statement reads:

“The anonymized dataset supporting the findings of this study is publicly available as Supporting Information. The dataset includes the variables analyzed in this study, comprising sociodemographic, environmental, territorial, and health-related information, as well as the corresponding codebook describing variable definitions, categories, and coding procedures used in the statistical and machine-learning analyses. All data were fully anonymized prior to sharing to ensure participant confidentiality and compliance with ethical standards for research involving vulnerable populations.”

The public file and its DOI were verified against the final analytical dataset before resubmission. Thus, the change from the initial statement reflects the preparation and verification of an anonymized minimum dataset, rather than a change in the ethical approval or an assumption that the original identifiable database could be shared without restriction.

Dataset DOI: https://doi.org/10.1371/journal.pntd.0014078.s001

Editor comment 6

Please ensure that the limitations explicitly discuss information bias associated with self-reported variables, selection bias related to community-based recruitment, and the limited number of communities included in contextual analyses and its implications for generalization.

Response

We agree and have substantially expanded the limitations section in the Discussion, lines 485-527. Regarding information bias, we now explain that healthcare need, receipt of care, and problem resolution were self-reported using a 12-month reference period. These measures may be affected by recall error, variation in individual interpretation, and social-desirability bias. We also state that these processes may have resulted in outcome misclassification and that, if reporting differed according to socioeconomic characteristics, community, previous service experiences, or interviewer interaction, the observed associations could have been attenuated or exaggerated.

Regarding selection bias, we now explicitly describe recruitment as community-based and non-probabilistic. Residents were included according to their presence, availability, and willingness to participate during fieldwork. We explain that this process may have overrepresented residents with greater availability or community engagement and underrepresented workers, mobile residents, people in less accessible areas, and individuals unwilling to participate. We further clarify that the sample-size calculation does not guarantee population representativeness.

Regarding contextual analyses, we now state that only nine communities were included and that their sample sizes were markedly unequal. Some communities contained very few participants, and analyses restricted to communities with at least 10 observations involved an even smaller number of aggregate units. Consequently, ecological correlations, variance components, and ICC estimates may be unstable and sensitive to individual communities. We explicitly state that community-level associations cannot be transferred directly to individual-level relationships and should be regarded as descriptive and hypothesis-generating.

We clarify that the findings may not be generalizable to other quilombola, urban, Indigenous, or Amazonian populations with different health-service networks, mobility conditions, and sociocultural characteristics.

REVIEWER 1

Reviewer 1 - Comment 1

Misalignment between the study objective and study design. The manuscript aims to “identify determinants” of healthcare access. However, given the cross-sectional design, this wording overstates the analytical capacity of the study.

Response

We thank the Reviewer and agree that the previous objective could be interpreted causally.

The objective was reformulated in the Abstract, lines 22–26, and the Introduction, lines 108–113, as follows:

“The present study aimed to examine individual, organizational, and territorial factors associated with self-reported healthcare access among quilombola rural and riverside communities in Santarém, Brazilian Amazon, using conventional statistical and machine-learning approaches.”

We replaced “identify determinants” with “examine factors associated with” because a cross-sectional study can estimate associations and describe patterns but cannot establish the temporal ordering required for causal interpretation.

The title, conclusion, cover letter, submission metadata, and response letter were also revised to use association-based terminology. In addition, the limitations and conclusion now explicitly state that the results do not establish temporal or causal relationships.

Reviewer 1 - Comment 2

Conceptual weakness of the primary outcome. The outcome is based on self-reported receipt of care when needed, which may conflate need, demand, and access and is subject to recall and reporting biases.

Response

We thank the Reviewer and agree that our previous description did not sufficiently distinguish the theoretical construct of healthcare access from its operational measurement in this study.

The primary outcome was redefined in the Methods, lines 162-179, as self-reported realized access: “The primary outcome was healthcare access operationalized as self-reported realized access, defined as having received healthcare when the participant perceived that care was needed during the previous 12 months.”

We now explicitly state that this measure captures only the successful receipt of care following a perceived need. It should not be interpreted as a comprehensive measure of healthcare accessibility and does not distinguish all stages between objective health need, perceived need, care-seeking, service availability, utilization, continuity, or adequacy.

We also explain that territorial mobility, transportation, primary-care organization, referral pathways, and health-system responsiveness were examined as contextual

Attachment Submitted filename: Response to Reviewers_L.docx

# Editor report: Decision Letter 2

**DOI:** 10.1371/journal.pone.0355489.r005

**Authors:** Angelica Miranda

22 Jul 2026

Healthcare Access Inequalities in Quilombola Rural and Riverside Communities in the Brazilian Amazon: Associations with Social, Organizational, and Territorial Factors

PONE-D-26-10264R2

Dear Dr. Valentim,

We’re pleased to inform you that your manuscript has been judged scientifically suitable for publication and will be formally accepted for publication once it meets all outstanding technical requirements.

Within one week, you’ll receive an e-mail detailing the required amendments. When these have been addressed, you’ll receive a formal acceptance letter and your manuscript will be scheduled for publication.

An invoice will be generated when your article is formally accepted. Please note, if your institution has a publishing partnership with PLOS and your article meets the relevant criteria, all or part of your publication costs will be covered. Please make sure your user information is up-to-date by logging into Editorial Manager at Editorial Manager® and clicking the ‘Update My Information' link at the top of the page. For questions related to billing, please contact billing support.

If your institution or institutions have a press office, please notify them about your upcoming paper to help maximize its impact. If they’ll be preparing press materials, please inform our press team as soon as possible -- no later than 48 hours after receiving the formal acceptance. Your manuscript will remain under strict press embargo until 2 pm Eastern Time on the date of publication. For more information, please contact onepress@plos.org.

Kind regards,

Angelica Espinosa Miranda, M.D., Ph.D.

Academic Editor

PLOS One

# Editor report: Acceptance letter

**DOI:** 10.1371/journal.pone.0355489.r006

**Authors:** Angelica Miranda

PONE-D-26-10264R2

PLOS One

Dear Dr. Valentim,

I'm pleased to inform you that your manuscript has been deemed suitable for publication in PLOS One. Congratulations! Your manuscript is now being handed over to our production team.

At this stage, our production department will prepare your paper for publication. This includes ensuring the following:

* All references, tables, and figures are properly cited

* All relevant supporting information is included in the manuscript submission,

* There are no issues that prevent the paper from being properly typeset

You will receive further instructions from the production team, including instructions on how to review your proof when it is ready. Please keep in mind that we are working through a large volume of accepted articles, so please give us a few days to review your paper and let you know the next and final steps.

Lastly, if your institution or institutions have a press office, please let them know about your upcoming paper now to help maximize its impact. If they'll be preparing press materials, please inform our press team within the next 48 hours. Your manuscript will remain under strict press embargo until 2 pm Eastern Time on the date of publication. For more information, please contact onepress@plos.org.

You will receive an invoice from PLOS for your publication fee after your manuscript has reached the completed accept phase. If you receive an email requesting payment before acceptance or for any other service, this may be a phishing scheme. Learn how to identify phishing emails and protect your accounts at https://explore.plos.org/phishing.

If we can help with anything else, please email us at customercare@plos.org.

Thank you for submitting your work to PLOS ONE and supporting open access.

Kind regards,

PLOS ONE Editorial Office Staff

on behalf of

Dr. Angelica Espinosa Miranda

Academic Editor

PLOS One
